# Deacetylation of TALDO1 by HDAC6 promotes glycolysis and nasopharyngeal carcinoma progression through a moonlighting function

**DOI:** 10.1038/s41419-025-08057-2

**Published:** 2025-10-21

**Authors:** Xingzhi Peng, Peijun Zhou, Kun Zhang, Likang Chen, Min Tang, Qin Zhou, Jinwu Peng, Lifang Yang

**Affiliations:** 1 Department of Oncology, Key Laboratory of Carcinogenesis and Cancer Invasion of Ministry of Education, National Clinical Research Center for Geriatric Disorders, Xiangya Hospital, Central South University, Changsha, China; 2 Cancer Research Institute, Xiangya School of Basic Medicine Sciences, Central South University, Changsha, China; 3 Department of Pathology, Xiangya Hospital, Central South University, Changsha, China; 4 Department of Pathology, Xiangya Changde Hospital, Changde, China

**Keywords:** Acetylation, Cancer metabolism

## Abstract

Aberrant metabolic enzymes drive glucose metabolism reprogramming, which plays a crucial role in tumor malignancy and metastasis. Protein acetylation is one of the key regulatory mechanisms of metabolic enzyme function, yet its precise role requires further clarification. In the present study, we reported that the deacetylation and low expression of transaldolase 1 (TALDO1) mediated by HDAC6 weakened the inhibitory effect of TALDO1 on tumor proliferation and metastasis in nasopharyngeal carcinoma (NPC). Mechanistically, highly expressed HDAC6 induced lysine 7 (K7) deacetylation of TALDO1, which could inhibit SMURF1-mediated K63-linked ubiquitination, thus reducing the protein stability of TALDO1. Notably, TALDO1 deacetylation inhibited its nuclear translocation and interaction with BRCA1, thereby reducing the inhibition of c-Myc transcriptional activation, promoting the expression of HK2/LDHA/PDK1, and further enhancing glycolysis independent of TALDO1 enzyme activity. This research elucidated the regulatory mechanism of TALDO1 from the perspective of acetylation modification, clarified the moonlighting functions of TALDO1 in metabolic reprogramming, and provided novel biomarkers and intervention strategies, such as HDAC inhibitors, for the clinical treatment of NPC.

## Introduction

Nasopharyngeal carcinoma (NPC) is a common malignant tumor of the head and neck in Southeast Asia and South China. With the innovation and development of radiation and combined therapy strategies, local NPC lesions have been effectively controlled [1, 2]. However, recurrence and distal metastases remain the main cause of treatment failure and death for NPC patients [3]. Therefore, uncovering the molecular mechanisms of NPC malignant progression and metastasis is necessary to guide to develop more effective treatment strategies.

Abnormal metabolism is a hallmark of tumors, with various pathways of glucose metabolism such as glycolysis, oxidative phosphorylation, and the pentose phosphate pathway (PPP) being reprogrammed to provide energy and biomolecules, maintain redox stability, and promote tumor proliferation and metastasis [4–6]. Studies have shown that aberrant expression and activity of metabolic enzymes within tumors are key drivers of metabolic reprogramming [5]. For example, the elevated HIF-1α upregulated the transcription and expression of pyruvate dehydrogenase (PDK1), promoted the conversion of pyruvate to lactate, thereby enhancing the glycolytic phenotype of metastatic cells and subsequently facilitating liver metastasis of breast cancer cells [7]. Glutamine deficiency promoted the palmitoyl transferase ZDHHC18 to mediate the palmitoylation of the tricarboxylic acid (TCA) cycle enzyme malate dehydrogenase 2 (MDH2), which enhanced the enzyme activity and mitochondrial respiration, and then promoted ovarian cancer cell proliferation [8]. O-GlcNAcylation of the PPP rate-limiting enzyme glucose-6-phosphate dehydrogenase (G6PD) enhanced its enzyme activity, further promoted synthesis of nucleotide and lipid precursors and reduced antioxidant defense equivalents, thus promoting the cell proliferation and survival of lung cancer [9]. However, the mechanisms underlying metabolic enzymes regulation during cancer malignant progression and metastasis are far from being clarified.

Protein acetylation is a post-translational modification that is reversibly regulated by acetyltransferases and deacetylases [10]. Acetylation modification is involved in gene transcription regulation through modulating histone acetylation status [11]. Notably, non-histone protein acetylation affects protein functions through diverse mechanisms, including the regulation of protein stability, enzymatic activity, subcellular localization, and protein-protein interactions [12]. For example, lysine 5 (K5) acetylation negatively regulated lactate dehydrogenase A (LDHA) enzyme activity and inhibited the proliferation and migration of pancreatic cancer [13]. PCAF-mediated acetylation of ATP-citrate lyase (ACLY) increased its stability by preventing ubiquitination degradation, and then enhanced de novo lipid synthesis, thereby promoting cell proliferation and tumor growth [14]. HDAC4-mediated deacetylation of glutaminase (GAC) at K311 promoted the interaction between TRIM21 and GAC, thereby facilitating K63-linked ubiquitination of GAC, which suppressed its enzymatic activity, consequently promoting the progression of lung cancer [15]. SIRT6-mediated deacetylation of pyruvate kinase M2 (PKM2) inhibited its nuclear translocation and made it lose the transcriptional coactivator function of oncogenes, thus inhibiting the proliferation and metastasis of liver cancer [16]. These studies indicate that acetylation modification is involved in the regulation of metabolic enzymes to impact their roles in tumor progression and metastasis [17].

Previously, we used tandem mass tags (TMT)-based quantitative acetylation proteomics to analyze differentially modified proteins in NPC cells [18]. In this study, low acetylated and expressed transaldolase 1 (TALDO1) was further clarified, and revealed that the K7 deacetylation of TALDO1 could suppress its protein stability and nuclear localization. The results also showed that the moonlighting function of TALDO1 induced by K7 deacetylation promoted glycolysis, thereby enhancing the proliferation and metastasis of NPC.

## Materials and methods

### Clinical samples

Paraffin-embedded NPC tissue microarrays (containing 126 cases of NPC and 14 cases of corresponding adjacent tissues) were purchased from Superboitek (NPC 1401, Shanghai, China), and the follow-up data and clinical details are shown in Table S1. A total of 35 primary NPC formalin-fixed paraffin-embedded tumor samples were retrospectively collected from Xiangya Hospital, Central South University (Changsha, China) [18]. The 82 serum samples of NPC patients were obtained from Xiangya Hospital, Central South University, and the clinicopathological information for these patients is summarized in Table S2.

### Plasmids and transfection

Blank vectors such as pcDNA3.1 (VT1001) and PGEX-4T-1 (VT1253) were purchased from Youbio (Changsha, China). pcDNA3.1-Flag (#20011), pCDH-CMV (#167463), pcDNA3.1-His (#52534), and pLKO.1 (#8453) were obtained from Addgene. pLVX-shRNA1 (632177) was purchased from Clontech (Mountain View, CA). The pCMVβ-p300-Myc (#30489), pcDNA3β-Flag-CBP-HA (#32908), pRK5-HA-Ubiquitin-WT (#17608), pRK5-HA-Ubiquitin-K48 (#17604), pRK5-HA-Ubiquitin-K63 (#17606), and pBABEpuro-HA-BRCA1 (#14999) plasmids were obtained from Addgene. pcDNA3.1-GCN5-His (40507), pcDNA3.1-PCAF-His (40506), and pcDNA3.1-TIP60-His (40505) plasmids were purchased from Youbio. The luciferase reporter vector pGL3-Basic (E1751) and Renilla luciferase reporter gene control vector pRL-TK (E2241) were obtained from Promega (Madison, WI). pcDNA3-HDAC6-DN-Flag plasmid was provided by Professor Wei Liu (School of Medicine, Zhejiang University) and pCDH-CMV-c-Myc was provided by Professor Hongjuan Cui (Medical Research Institute, Southwest University). Regarding plasmid construction, a full-length TALDO1 coding sequence(CDS) was respectively cloned into pcDNA3.1, pcDNA3.1-Flag and pCDH-CMV vectors to generate pcDNA3.1-TALDO1, pcDNA3.1-TALDO1-Flag and pCDH-CMV-TALDO1, pcDNA3.1-TALDO1 and pcDNA3.1-TALDO1-Flag were used for transient transfections, while pcDH-CMV-TALDO1 was used for stable cell line construction. The CDS of HDAC4, HDAC5, HDAC6, SMURF1, and SYVN1 were cloned into pcDNA3.1-His to generate pcDNA3.1-HDAC4-His, pcDNA3.1-HDAC5-His, pcDNA3.1-HDAC6-His, pcDNA3.1-SMURF1-His, and pcDNA3.1-SYVN1-His plasmids. pcDNA3.1-His vector was digested with ApaI (1604, Takara, Tokyo, Japan), and HDAC6-DN coding sequence from HDAC6-DN-Flag plasmid was inserted into the digested vector to obtain pcDNA3.1-HDAC6-DN-His plasmid. The TALDO1-WT/K7R/K7Q sequences were cloned into the PGEX-4T-1 vector to create GST-TALDO1-WT/K7R/K7Q plasmids. The HK2, PDK1, and LDHA promoter sequences were gained from PCR amplification and cloned into the pGL3-basic vector to generate pGL3-HK2/PDK1/LDHA plasmids. The construction of expression plasmids using the Recombination Kit (C112-02, Vazyme, Nanjing, China) and the primers are listed in Table S3-1. The pcDNA3.1-TALDO1(K7R)-Flag and pcDNA3.1-TALDO1(K7Q)-Flag mutants were generated from pcDNA3.1-TALDO1-Flag. pCMVβ-p300.DY-myc mutant was generated from pCMVβ-p300-Myc. K48R and K63R mutants were generated using pRK5-HA-Ubiquitin-WT. Point mutations were introduced using a point mutation kit (C215, Vazyme) and primers for the construction of mutated plasmids are listed in Table S3-2. The shRNAs targeting TALDO1 and HDAC6 were cloned into pLVX-shRNA1, while shRNAs targeting BRCA1 and c-Myc were cloned into pLKO.1. The primers for the construction of shRNA plasmids are listed in Table S3-3, and siRNA sequences for HDAC4 and HDAC5 are listed in Table S3-4.

Lipofectamine 2000 (12566014, Invitrogen, Carlsbad, CA) was used for all plasmid transfections. Lipofectamine 3000 (L3000075, Thermo Fisher Scientific, Waltham, MA) was used for all siRNA transfections. Transfection experiments were performed according to the manufacturer’s instructions.

### Cell culture

All NPC cell lines (HK1, SUNE1, HONE1, 5-8F, 6-10B, C666-1), normal human nasopharyngeal epithelial cell lines (NP69, NP460), and 293T cells were sourced from the Cell Center of Central South University. NP69 cells were cultured in keratinocyte serum-free medium (17005042, Gibco, Grand Island, NY). NP460 cells were cultured in EpiLife Medium (MEPI500CA, Gibco). HONE1, 5-8F, 6-10B, and C666-1 cells were maintained in RPMI 1640 (C11875500BT, Gibco) with 10% bovine calf serum (BCS) (B7446, Sigma-Aldrich, St. Louis, MO). SUNE1 and HK1 cells were cultured in RPMI 1640 supplemented with 10% fetal bovine serum (FBS) (F8687, Sigma-Aldrich). 293T cells were cultured in DMEM (C11995500BT, Gibco) with 10% BCS. All cells were incubated at 37 °C with 5% CO_2_.

The 293T cells were co-transfected with shRNA or overexpression plasmids and lentivirus packaging plasmids (gag/pol, #14887, pRSV-Rev, #12253, pCMV-VSV-G, #8454, Addgene) using Lipofectamine 2000 to produce recombinant lentivirus. After 48 h, the viral supernatant was collected and used to infect NPC cells, which were selected with puromycin (ST551, Beyotime, Shanghai, China).

### Antibodies and reagents

Primary antibodies used were TALDO1 (12376-1-AP), Flag-tag (80010-1-RR), HDAC4 (17449-1-AP), HDAC5 (16166-1-AP), HDAC6 (12834-1-AP), His-tag (66005-1-Ig), HA-tag (66006-2-Ig), Myc-tag (16286-1-AP), p300 (20695-1-AP), BRCA1 (22362-1-AP), and c-Myc (10828-1-AP) from Proteintech (Chicago, IL); SMURF1 (sc-100616) and SYVN1 (sc-293484) from Santa Cruz Biotechnology (Santa Cruz, CA); Actin (AC026) from Abclonal (Wuhan, China). K63-Ub (#5621), K48-Ub (#8081), PDK1 (#3062), LDHA (#3582), PKM2 (#4053), and Histone H3 (#4499) from Cell Signaling Technology (Danvers, MA); HK2 (ab209847), and Acetyl Lysine (ab21623) from Abcam (Cambridge, MA). LBH589 (S1030), PXD101 (S1085), 3-MA (S2767), and CAY10603 (S7596) from Selleck Chemicals (Houston, TX); LMK-235 (HY-18998), MS-275 (HY-12163), MG132 (HY-13259), CHX (HY-12320), CHQ (HY-17589A), and 2-DG (HY-13966) from MedChemExpress (Monmouth Junction, NJ).

### Western blot analysis and immunoprecipitation (IP)

Cell samples were lysed in IP buffer (P0013, Beyotime) with protease inhibitor cocktail (4693116001, Roche, Basel, Switzerland). Protein concentration was measured using BCA assay kit (AR0197, Boster, Wuhan, China). 50 μg of cell lysate was separated by SDS-PAGE and transferred to a PVDF membrane (IPVH00010, Merck Millipore, Billerica, MA). After blocking with 5% milk for 1 h, the membrane was incubated with primary antibody at 4 °C overnight. Following TBST washes, the membrane was incubated with peroxidase-linked secondary antibody for 1 h and then detected with luminol substrate (32209, Thermo Fisher Scientific). Proteins were visualized using the Bio-Rad ChemiDoc XRS system (Hercules, CA). As for IP, cell lysates were prepared using IP buffer with protease inhibitor cocktail. 30 μL of magnetic beads (B23201, Selleck Chemicals) were added to 500 μL of lysate and incubated for 2 h at 4 °C with rotation. After bead removal, the supernatant was incubated overnight at 4 °C with 2 μg of antibody. The mixture was then incubated with 30 μL of magnetic beads for 2 h at 4 °C with rotation. Bead-antibody-protein complexes were washed three times with cold IP buffer and boiled for 10 min with 5× SDS-PAGE loading buffer for subsequent western blot.

### Quantitative PCR (qPCR)

Total RNA was extracted with TRIzol reagent (15596026, Thermo Fisher Scientific). 2 μg RNA was reverse transcribed to cDNA using a Reverse Transcription Kit (K1621, Thermo Fisher Scientific). qPCR was performed in triplicate with SYBR Green (4309155, Life Technologies Corporation, Gaithersburg, MD) on a CFX Connect Real-Time PCR System (Bio-Rad). Relative mRNA expression was calculated using the 2^−ΔΔCT^ method with β-actin as the reference. Primers are listed in Table S3-5.

### In vivo ubiquitination assay

293T cells were co-transfected with the indicated plasmids. After 48 h, cells were treated with 20 μM MG132 for 6 h and lysed in IP buffer with protease inhibitor cocktail. Lysates were boiled for 10 min and then diluted with 900 μL of buffer (10 mM Tris-HCl, pH 8.0, 150 mM NaCl, 2 mM EDTA, 1% Triton X-100) and incubated at 4 °C for 30 min with rotation. After centrifugation, the supernatant was incubated overnight at 4 °C with anti-Flag or anti-TALDO1 antibodies, and then with 30 μL of magnetic beads for 2 h at 4 °C with rotation. Bead-antibody-protein complexes were washed three times with cold IP buffer, boiled with 5× SDS-PAGE loading buffer, and analyzed by immunoblotting using anti-K63Ub, anti-K48Ub, or anti-HA antibodies.

### Immunofluorescence (IF)

HK1 cells were transfected with the indicated plasmids for 48 h. Cells were fixed with 4% paraformaldehyde for 20 min, washed with 1× PBS, and permeabilized with 0.3% Triton X-100 in PBS for 15 min. After blocking with 5% goat serum for 60 min at 37 °C, cells were incubated overnight at 4 °C with primary antibodies diluted in 5% goat serum: Flag-tag (1:200, 80010-1-RR, Proteintech); His-tag (1:200, 66005-1-Ig, Proteintech); Flag-tag (1:200, F1804, Sigma-Aldrich); Myc-tag (1:200, 16286-1-AP, Proteintech). Following washes, cells were incubated with secondary antibodies in 5% goat serum for 2 h at 37 °C: Anti-Rabbit-CF 488 A (1:2000, SAB4600234, Sigma-Aldrich), and Anti-Mouse-Alexa Fluor 594 (1:2000, A-11005, Thermo Fisher Scientific). DNA was stained with DAPI. Cells were washed with 1× PBS and imaged using a Confocal Microscope (TCS SP8, Leica, Wetzlar, Germany).

### ELISA examination

The concentrations of TALDO1 in NPC patient clinical serum samples were tested using an ELISA kit (HM11727, Bioswamp, Wuhan, China) according to the manufacturer’s protocol.

### CCK-8 assay

Cell proliferation activity was measured by the CCK-8 kit (C0005, TargetMol, Boston, MA). 1 × 10^3^ NPC cells were cultured in 96-well plates and allowed to attach overnight at 37 °C. Then, at a certain time, 10 μL CCK-8 was added to each well. After being cultured at 37 °C for 2 h, the absorbance was measured at 450 nm on a plate reader (ELx800, Biotek, Winooski, VT).

### Colony formation assay

1 × 10^3^ NPC cells were seeded in each well of 6-well plates. After 10 to 14 days of culture, cells were washed three times with PBS, then stained with 0.05% crystal violet (V5265, Sigma-Aldrich). Colonies in each well were scanned, and the data were analyzed.

### Apoptosis detection

Cells of appropriate density were harvested, resuspended in binding buffer, and then incubated with Annexin V-FITC and PI (KGA1102, Keygen, Jiangsu, China) for 10 min, and the cell apoptosis was detected by flow cytometry (MoFlo XDP, Beckman Coulter, Miami, FL) and analyzed with FlowJo software.

### Transwell assay and wound healing

Transwell invasion assays were performed using 24-well Cell Culture Inserts (353097, Corning, NY) coated with Matrigel (354234, Corning). After 24 h of incubation at 37 °C, non-migrated cells in the upper chamber were removed. Cells were then fixed with 4% paraformaldehyde for 15 min, stained with crystal violet (V5265, Sigma-Aldrich), and counted from three random fields. For wound-healing assays, cells were cultured in 24-well plates and wounds were created using a 10 µL pipette tip. Cell migration was monitored under a microscope (DMI 3000B, Leica, Wetzlar, Germany), and the wound-healing rate was analyzed using ImageJ software.

### Recombinant TALDO1 protein and enzyme activity assay

GST-TALDO1-WT/K7R/K7Q fusion proteins were expressed in *E. coli* BL21. BL21 were cultured at 37 °C to an OD600 of 0.6, then induced overnight with 0.1 mM IPTG at 18 °C. Proteins were purified using the GST-tag Protein Purification Kit (P2262, Beyotime). For the enzyme activity assay, GST tags were cleaved from the fusion proteins.TALDO1 enzyme activity was tested in the presence of 3.2 mM D-fructose 6-phosphate, 0.2 mM erythrose-4-phosphate, 0.1 mM NADH, 5 mM EDTA, and 10 μg of α-glycerophosphate dehydrogenase/triosephosphate isomerase at a 1:6 ratio in 40 mM triethanolamine at room temperature by continuous absorbance reading at 340 nm for 20 min [19].

### Targeted metabolomics

1 × 10^6^ SUNE1-Vec or SUNE1-TALDO1 stably expressing cells were extracted for targeted metabolites as previously described with slight modification [20]. Then the cells were sent to Metware (Wuhan, China) for targeted metabolomics. Each group included six parallel samples. Results were normalized to the BCA protein of the cells in each sample. log2FC (fold change) ≥ 1.5 and FDR-adjusted *p* < 0.05 were used to screen for differential metabolic substrates. Orthogonal partial least squares discriminant analysis (OPLS-DA) was used to assess the overall data structure and group discrimination. Based on the OPLS-DA model, the Variable Importance in Projection (VIP) scores (VIP ≥ 1) derived from the multivariate model were used to identify significant differential metabolites between the WT and KR groups. Specifically, the screening criteria were as follows: log2FC (fold change) ≥ 1.5, VIP ≥ 1, and FDR-adjusted *p* < 0.05.

### Lactate production assay

The cell culture medium of NPC cells was collected, and the lactate concentration was determined using a lactate assay kit (MAK064, Sigma-Aldrich) according to the manufacturer’s protocol.

### Glucose uptake assay

The cell culture medium of NPC cells was collected, and the levels of glucose consumption were examined according to the instructions of the glucose uptake colorimetric assay kit (MAK083, Sigma-Aldrich).

### Seahorse assay

Extracellular Acidification Rate (ECAR) was measured by using a Seahorse XF96 instrument (Seahorse Biosciences, Billerica, MA) according to the manufacturer’s protocol. Cells were counted and seeded into the Seahorse 96-well plate at a density of 1 × 10^4^ cells/well, and cultured for 12 h. After that, 10 mM glucose, 1 μM oligomycin and 50 mM 2-DG were added to different ports of the Seahorse cartridge. Results were normalized to the BCA protein of the cells in each well.

### Liquid chromatography mass spectrometry (LC-MS)

HK1 cells were transfected with Flag-TALDO1 plasmid for 48 h, and the total protein was lysed with IP buffer containing protease inhibitor cocktail. Subsequently, IP experiment was performed with Flag-tag antibody, and magnetic beads-antibody-protein complexes were sent to Huaying Biotechnology (Shanghai, China) for LC-MS/MS analysis using the Orbitrap Fusion Lumos Tribrid mass spectrometer (Thermo Scientific).

### Nuclear/cytoplasmic fractionation

Nuclear and cytoplasmic lysates were prepared using the NE-PER Nuclear and Cytoplasmic Extraction Reagent kit (78833, Pierce, Rockford, IL) according to the manufacturer’s protocol.

### Dual luciferase reporter assay

pGL3-HK2/PDK1/LDHA promoters, pRL-TK and other indicated plasmids were co-transfected into SUNE1 cells. After 48 h of culture, the Dual Luciferase Reporter Assay Kit (RG027, Beyotime) was used according to the manufacturer’s protocol. Relative luciferase activity was calculated by normalizing to the quantified pRL-TK activity.

### Animal experiments

BALB/c female nude mice were randomly grouped. To verify the role of TALDO1 in NPC tumorigenesis, 5 × 10^6^ SUNE1-Vec or SUNE1-TALDO1 cells suspended in 100 μL of PBS were injected subcutaneously into five-week-old mice to establish xenografts (*n* = 5). To investigate the role of HDAC6 in NPC progression, 5 × 10^6^ SUNE1-shVec or SUNE1-shHDAC6 cells were similarly injected (*n* = 5). Tumor size was measured every two days and calculated using the formula V = a × b^2^/2, where a and b were the longest and shortest diameters, respectively. To assess the role of TALDO1 in NPC metastasis, 1 × 10^6^ SUNE1-Vec or SUNE1-TALDO1 cells in 50 μL of PBS were injected into the tail vein of five-week-old mice (*n* = 5). Additionally, to explore the effect of CAY10603 on NPC metastasis in vivo, 1 × 10^6^ SUNE1 cells were injected into the tail vein of five-week-old mice (*n* = 5), followed by treatment with CAY10603 (20 mg/kg, intraperitoneally, every three days for one month). After 6–8 weeks, the mice were sacrificed, and lung tissues were collected for Immunohistochemistry (IHC) and hematoxylin and eosin (H&E) staining.

### IHC

Paraffin-embedded tumor tissues were sectioned into 5–10 μm slices and deparaffinized. Antigen retrieval was performed in 10 mM citrate buffer (pH 6.0) at 95 °C for 30 min. After quenching endogenous peroxidase using a universal two-step kit (PV-9000, ZSGB-BIO, Beijing, China) and blocking with 5% normal goat serum, slices were incubated with primary antibodies followed by peroxidase-linked secondary antibodies. Sections were displayed using a DAB Kit (ZLI-9017, ZSGB-BIO) and re-stained with hematoxylin (E607317, Sangon Biotech, Shanghai, China). Protein expression was semi-quantitatively evaluated using the method previously described [21].

### Bioinformatics analysis

The TCGA-HNSC database and GSE12452 database were used for TALDO1 mRNA level analysis. The TCGA-HNSC database was used for survival curve analysis. The UniProt database (https://www.uniprot.org/) was used to analyze the conservation of potential acetylation sites in TALDO1.

### Statistical analysis

According to statistical parameters, each experiment was repeated at least three times independently. GraphPad Prism 8 was used for statistical analysis. Data were expressed as the mean ± SD and analyzed using two-tailed t-tests, whereas for Kaplan–Meier analysis, the Log-rank (Mantel-Cox) test was used, and correlations were calculated using the parametric two-tailed Pearson correlation test. *p* < 0.05 (*), *p* < 0.01 (**) and *p* < 0.001 (***) were considered statistically significant.

## Results

### TALDO1 is deacetylated and downregulated in NPC

**Fig. 1:**
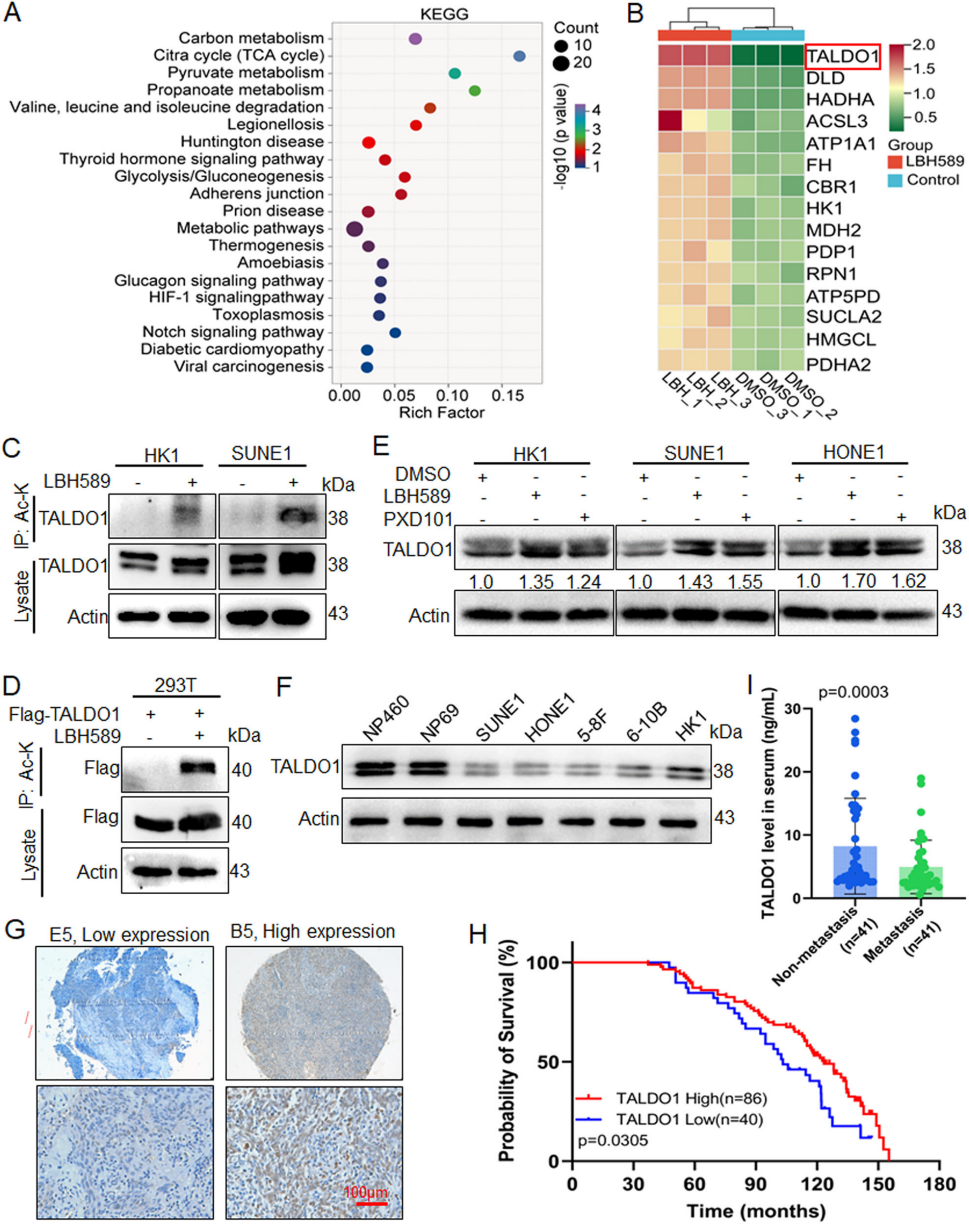
TALDO1 is deacetylated and downregulated in NPC. **A**, **B** LC-MS/MS assays in HK1 cells with HDACi LBH589 treatment (100 nM, 24 h). **A** KEGG pathways enrichment analysis of the upregulated acetylated non-histone proteins using Ingenuity Pathway Analysis (IPA). **B** The heatmap of the upregulated acetylated metabolism enzymes in the LBH589-treated groups compared with DMSO groups (Fold change > 1.5, *p*-value < 0.05). **C** After treatment with LBH589 (100 nM, 24 h) in HK1 and SUNE1 cells, TALDO1 acetylation was detected by IP with an anti-acetylated lysine antibody and western blot. Ac-K acetylated lysine. **D** After transfected Flag**-**TALDO1 in 293T cells and treating with LBH589, TALDO1 acetylation was examine by IP and western blot. **E** Western blot analysis of TALDO1 in HK1, SUNE1, and HONE1 cells treated with LBH589 (100 nM, 24 h) or PXD101 (50 nM, 24 h). **F** Western blot analysis of TALDO1 in NPC cell lines (SUNE1, HONE1, 5-8F, 6-10B and HK1) and two normal nasopharyngeal mucosal/epithelial cell lines (NP460 and NP69). IHC analysis of TALDO1 in NPC tissue microarrays. **G** The TALDO1 expression in NPC tissues by IHC staining (*n* = 126). **H** Overall survival rates in NPC patients with low or high TALDO1 expression assessed by Kaplan–Meier analysis. **I** TALDO1 protein levels in the serum of patients with metastatic or non-metastatic NPC were measured using the TALDO1 ELISA kit. Data were shown as the mean ± SD of at least three independent experiments.

Due to tumor suppressor molecules are usually deacetylated to lose their function in most tumors, histone deacetylase inhibitor (HDACi) can inhibit the malignant progression of tumors by restoring their acetylation [12, 22]. In order to investigate tumor suppressor proteins directly regulated by deacetylation in NPC, TMT-based acetylation quantitative proteomic analysis was performed in NPC HK1 cells treated with pan-HDACi LBH589 [18]. KEGG analysis of upregulated acetylated proteins showed significant enriched in cellular energy metabolic pathways, such as carbon metabolism, citrate cycle, pyruvate metabolism, and glycolysis/gluconeogenesis (Fig. 1A). Among them, 15 metabolic enzymes were upregulated acetylated after LBH589 treatment (Fold change > 1.5, *p* < 0.05) (Fig. 1B, Table S4). TALDO1 was the metabolic enzyme with the most significant upregulation of acetylation. IP experiments with a pan-acetylated lysine antibody confirmed increased TALDO1 acetylation and expression in LBH589-treated cells (Fig. 1C, D). Western blot analysis demonstrated elevated TALDO1 expression in NPC cells treated with LBH589 or another pan-HDACi PXD101 (Fig. 1E). Additionally, TALDO1 was lower expressed in NPC cells compared to normal nasopharyngeal cells (Fig. 1F). Consistently, analyses of TALDO1 mRNA expression using TCGA and GEO datasets showed that TALDO1 was lowly expressed in HNSC and NPC, while the mRNA levels were not significantly associated with prognosis in HNSC patients (Fig. S1). However, IHC staining analysis of NPC tissue microarrays showed that lower TALDO1 expression correlated with poorer overall survival (Fig.1G, H). These results suggest its protein expression may have more obvious biological functions and the importance of post-transcriptional modification of TALDO1. Further, recent studies have reported that TALDO1 protein could be detected in the serum of patients with colorectal cancer [23]. Therefore, ELISA analysis of NPC serum samples with and without metastasis showed lower TALDO1 in metastatic cases (Fig. 1I). In summary, these findings suggest that the reduction of TALDO1 protein expression in NPC may be due to deacetylation, and the decrease of TALDO1 may contribute to tumor progression and metastasis.

### TALDO1 inhibits proliferation and metastasis of NPC

**Fig. 2:**
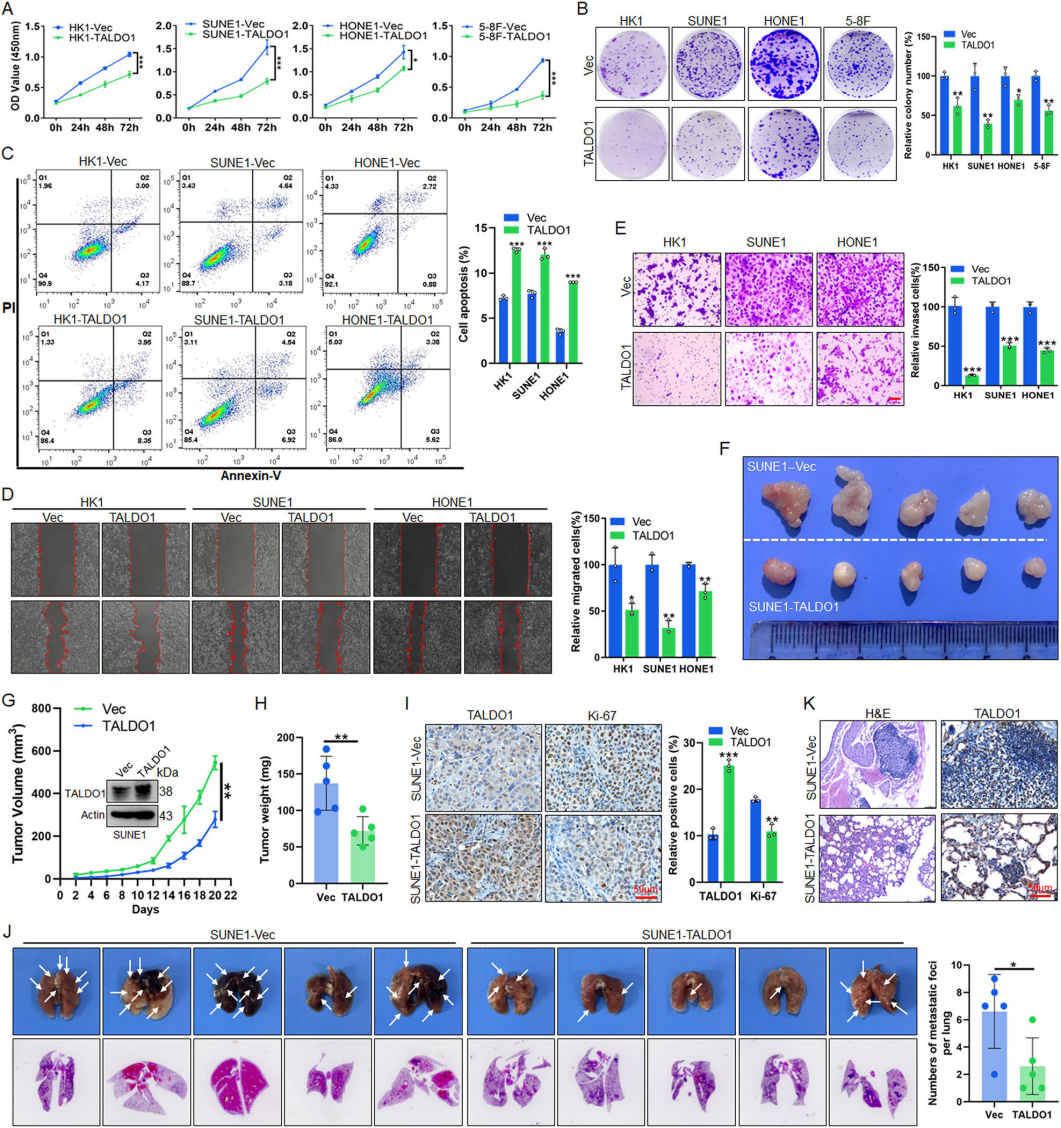
TALDO1 inhibited the proliferation and metastasis of NPC. **A**–**E** After transfections of pcDNA3.1-TALDO1 plasmid into NPC cells for 48 h. **A**, **B** CCK-8 assay and colony forming assay were performed to examine the cell proliferation. **C** The cell apoptosis was analyzed by flow cytometry. **D** Wound-healing assay was performed to detect cell migration. **E** Transwell assays were carried out to detect cell invasion. Scale bar, 50 μm. **F**–**I** After 5 × 10^6^ SUNE1-Vec and SUNE1-TALDO1 stable cells were injected subcutaneously into 5-week-old nude mice (*n* = 5), the tumor growth (**F**), tumor volume (**G**) and tumor weight (**H**) were monitored. **I** Representative images of IHC staining of TALDO1 and Ki-67 in xenograft tumor tissues. **J**, **K** After 1 × 10^6^ SUNE1-Vec and SUNE1-TALDO1 stable cells were injected into the lateral tail vein of 5-week-old nude mice (*n* = 5), **J** Images and quantitative data of lung metastatic foci, **K** Representative images of H&E staining of lung foci and IHC staining of TALDO1 in lung foci. Data were shown as the mean ± SD of at least three independent experiments. *p < 0.05 and **p < 0.01 and ***p < 0.001.

To investigate the function of TALDO1 in NPC, we constructed a TALDO1-overexpressing plasmid and confirmed its effect by qPCR and western blot (Fig. S2). CCK-8 (Fig. 2A) and colony formation assays (Fig. 2B) showed that TALDO1 overexpression significantly suppressed NPC cell proliferation. Flow cytometry indicated that overexpression of TALDO1 induced cell apoptosis (Fig. 2C), and wound-healing and Transwell assays demonstrated reduced cell migration and invasion after overexpression of TALDO1 (Fig. 2D, E). In xenograft experiments, tumor growth was significantly slower in mice injected with TALDO1-overexpressing SUNE1 cells (Fig. 2F–H), and IHC staining revealed a decrease in Ki-67 expression (Fig. 2I). Metastasis experiments showed fewer and smaller metastatic lung foci in the TALDO1-overexpressing group (Fig. 2J, K). These findings collectively suggest that TALDO1 inhibits NPC proliferation and metastasis in vitro and in vivo.

### HDAC6 mediates deacetylation of TALDO1

**Fig. 3:**
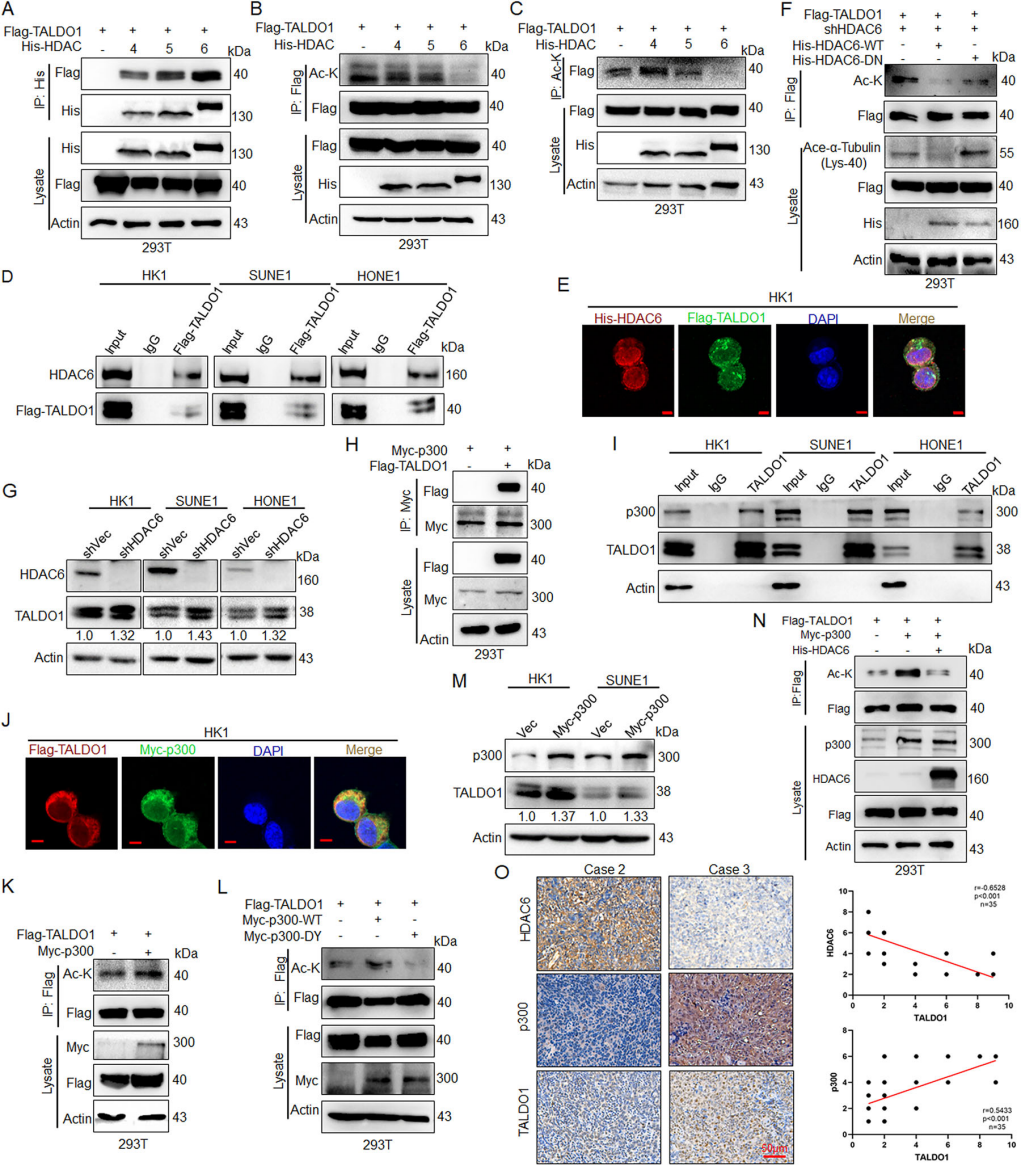
HDAC6 mediates deacetylation of TALDO1. **A**–**C** After indicated plasmids were transfected into 293T cells for 48 h, **A** Interaction between HDAC4/5/6 and TALDO1 was detected by IP and western blot. **B**, **C** TALDO1 acetylation was detected by IP and western blot. **D** After transfected Flag-TALDO1 in NPC cells, interaction between exogenous Flag-TALDO1 and endogenous HDAC6 was analyzed by IP and western blot. **E** Indicated plasmids were transfected into HK1 cells, and the colocalization of TALDO1 and HDAC6 was investigated by confocal microscopy. Scale bar, 5 μm. **F** Indicated plasmids were transfected into 293T cells, TALDO1 acetylation was detected by IP and western blot. **G** After established HK1, SUNE1 and HONE1 knockdown HDAC6 stable cell lines through transfected shHDAC recombinant lentivirus, western blot analysis of TALDO1 expression in HDAC6 stable knockdown NPC cells. **H** After co-transfected Flag-TALDO1 and Myc-p300 plasmids in 293T cells, and interaction between TALDO1 and p300 was detected by IP and western blot. **I** The endogenous interaction between TALDO1 and p300 was detected by IP and western blot. **J** After co-transfected Flag-TALDO1 and Myc-p300 plasmids in HK1 cells, the colocalization of TALDO1 and p300 was investigated by confocal microscopy. Scale bar, 5 μm. **K** After co-transfected Flag-TALDO1 and Myc-p300 plasmids in 293T cells, TALDO1 acetylation was detected by IP and western blot. **L** After co-transfected Flag-TALDO1 and Myc-p300 WT or DY (a catalytic inactivation mutant of p300) plasmids in 293T cells, TALDO1 acetylation was analyzed by IP and western blot. **M** Western blot analysis of TALDO1 in p300 overexpression NPC cells. **N** After co-transfected Flag-TALDO1 and Myc-p300 followed by transfection with His-HDAC6 expression plasmid for 48 h, TALDO1 acetylation was detected by IP and western blot. **O** The expression and correlation of TADLO1 and HDAC6 or p300 were analyzed by IHC staining of clinical NPC tissues. Sample size (*n* = 35), Pearson correlation coefficient (r) and *p*-value (two-tailed) are indicated.

To identify the potential deacetylase for TALDO1, two selective HDACi were used. The data showed that TALDO1 acetylation was significantly induced by LMK-235 (specific HDAC4/5/6/11/8 inhibitor) but not MS-275 (specific HDAC1/2/3 inhibitor), and LMK-235-treated NPC cells exhibited higher TALDO1 protein expression (Fig. S3A, B). IP assays in 293T cells with His-tagged HDAC4, 5, and 6 suggested that TALDO1 interaction with these deacetylases (Fig. 3A). However, only ectopic HDAC6 expression significantly induced TALDO1 deacetylation (Fig. 3B, C). Further, IP experiments in NPC cells transfected with Flag-TALDO1 confirmed TALDO1 coprecipitation with HDAC6 (Fig. 3D), and extensive colocalization of HDAC6 with TALDO1 was observed in HK1 cells (Fig. 3E). Consistent with this, deacetylase-dead HDAC6 (HDAC6-DN) [24] did not alter TALDO1 acetylation compared to wild type in shHDAC6 293T cells (Fig. 3F). Therefore, we established stable NPC cell lines with HDAC6 knockdown, and found that HDAC6 knockdown (Fig. 3G), but not HDAC4 or HDAC5 (Fig. S3C), increased TALDO1 protein in NPC cells.

To identify the acetyltransferase acting on TALDO1, we first investigated the interactions between TALDO1 and five common acetyltransferases. IP experiments showed that TALDO1 directly interacted with p300 in 293T cells (Fig. S4A), and p300 overexpression could promote TALDO1 protein expression (Fig. S4B). Further exogenous and endogenous IP experiments confirmed TALDO1 coprecipitation with p300 (Fig. 3H, I), and IF showed strong colocalization of two molecules (Fig. 3J). Consistent with this, IP experiments in 293T cells demonstrated that overexpression of p300-wild type (WT), but not acetyltransferase-deficient p300 (p300-DY) [25], induced TALDO1 acetylation (Fig. 3K, L). Furthermore, p300 overexpression increased TALDO1 protein in NPC cells (Fig. 3M). Importantly, our results showed that HDAC6 disrupted p300-mediated upregulation of TALDO1 acetylation (Fig. 3N). Additionally, IHC staining of NPC patient tissue samples showed a strong negative correlation between HDAC6 and TALDO1 expression, and a positive correlation between p300 and TALDO1 expression (Fig. 3O). These findings suggest that HDAC6-induced deacetylation decreases TALDO1 protein expression, whereas p300-mediated acetylation enhances TALDO1 expression.

### HDAC6 promotes proliferation and metastasis of NPC through TALDO1

**Fig. 4:**
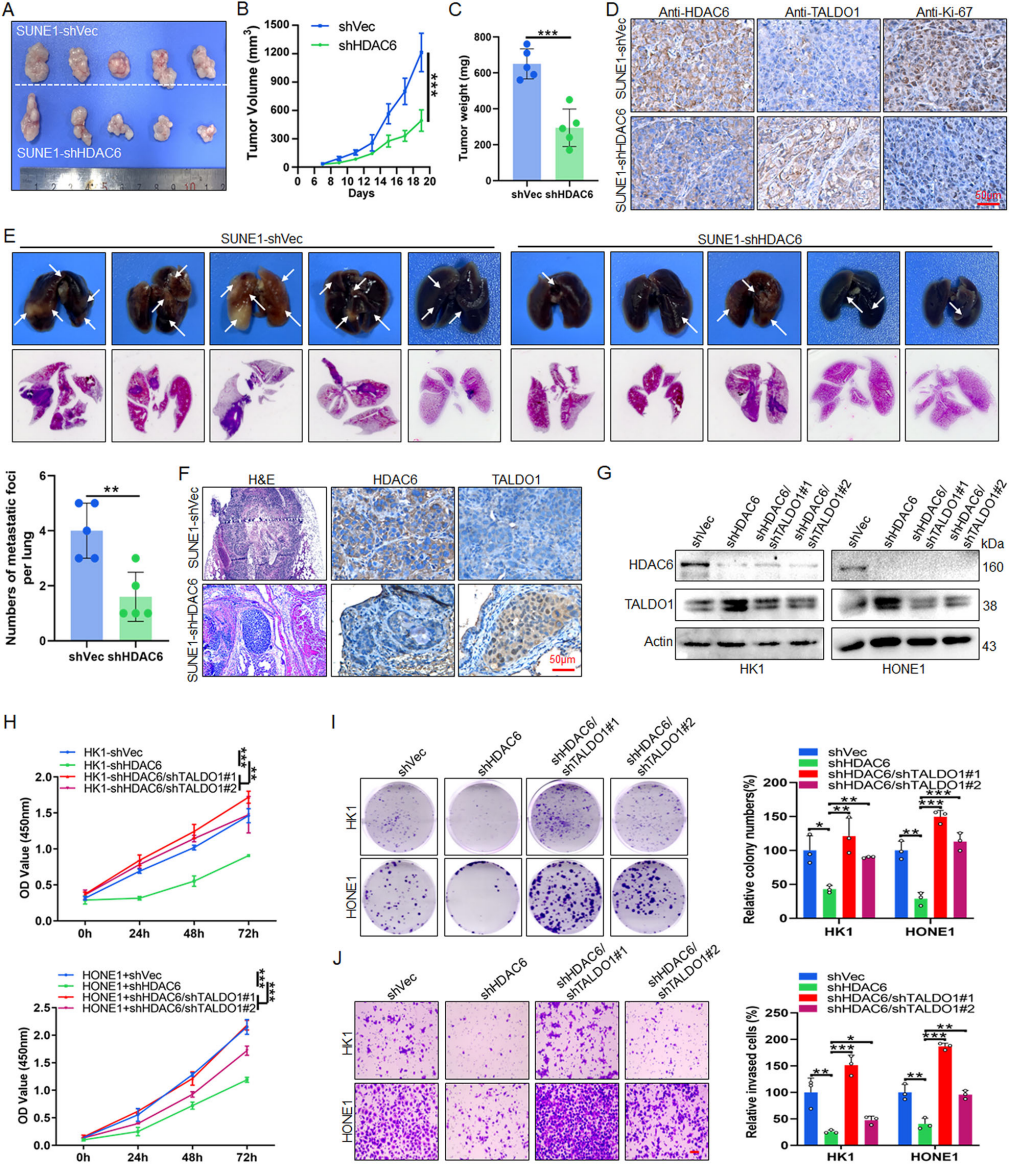
HDAC6 promotes the proliferation and metastasis of NPC through TALDO1. **A**–**C** After SUNE1-shVec and SUNE1-shHDAC6 stable cells were injected subcutaneously into 5-week-old nude mice (*n* = 5), the tumor growth (**A**), tumor volume (**B**) and tumor weight (**C**) were monitored. **D** Representative images of IHC staining of HDAC6, TALDO1 and Ki-67 in xenograft tumor tissues. **E**, **F** After SUNE1-shVec and SUNE1-shHDAC6 stable cells were injected into the lateral tail vein of 5-week-old female nude mice (*n* = 5), **E** images and quantitative data of lung metastatic foci, **F** representative images of H&E staining of lung foci and IHC staining of HDAC6 and TALDO1 in lung foci. **G** Western blot analysis of TALDO1 expression after TALDO1 knockdown in HK1-shHDAC6 and HONE1-shHDAC6 stable cells. **H**, **I** CCK-8 assays (**H**) and colony forming assay (**I**) were performed to examine the cell proliferation. **J** Transwell assays were carried out to detect cell invasion. Scale bar, 50 μm. Data were shown as the mean ± SD of at least three independent experiments. *p < 0.05 and **p < 0.01 and ***p < 0.001.

To investigate the function of HDAC6 in NPC, the expression of HDAC6 was examined, and qPCR and western blot data showed that HDAC6 was highly expressed in NPC cells (Fig. S5A, B). CCK-8, colony formation and Transwell assays indicated that HDAC6 knockdown significantly inhibited NPC cell proliferation and invasion (Fig. S5C–F). Further, xenograft experiments demonstrated that the tumor growth was significantly slower in the shHDAC6 group (Fig. 4A–C), and IHC staining showed a decrease in HDAC6 and Ki-67, while an increase in TALDO1 expression (Fig. 4D). Meanwhile, metastatic experiments indicated fewer lung metastatic foci in the shHDAC6 group, with reduced HDAC6 and increased TALDO1 expression in metastases (Fig. 4E, F). Consistently, NPC cells treated with the HDAC6-specific inhibitor CAY10603 exhibited a concentration-dependent increase in TALDO1 expression (Fig. S6A). Functional assays further showed that CAY10603 significantly inhibited NPC proliferation and invasion (Fig. S6B–D), and reduced metastasis in vivo (Fig. S6E, F). To determine whether HDAC6 affects NPC proliferation and invasion by inhibiting TALDO1, HK1 and HONE1 cells with double knockdown of HDAC6 and TALDO1 were generated (Fig. 4G). CCK-8 (Fig. 4H), colony formation (Fig. 4I) and Transwell assays (Fig. 4J) indicated that TALDO1 knockdown rescued the proliferation and invasion defects caused by HDAC6 silencing. Collectively, these results demonstrate that HDAC6 promotes NPC proliferation and metastasis by downregulating TALDO1.

### K7 deacetylation attenuates TALDO1 stability

**Fig. 5:**
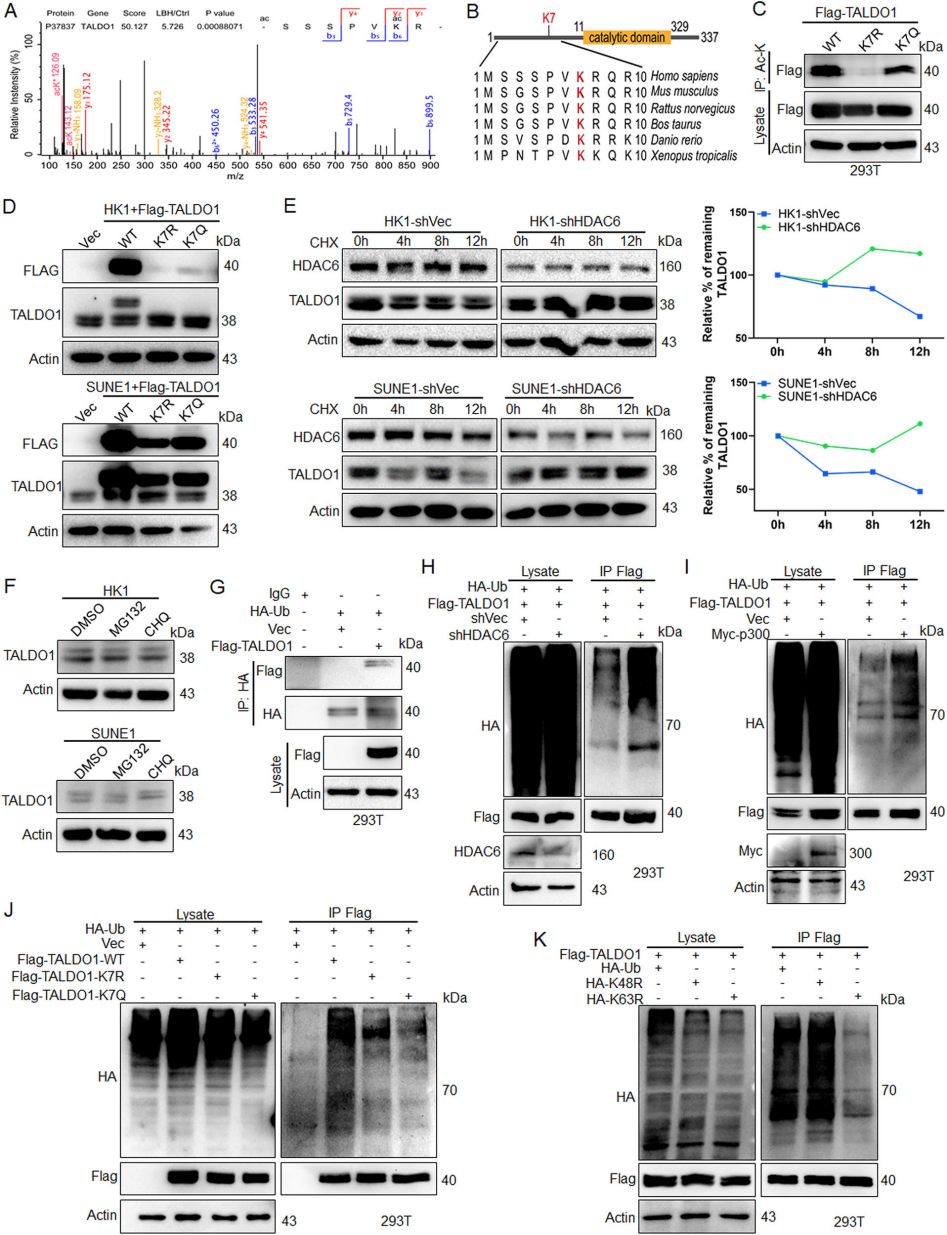
K7 deacetylation attenuates TALDO1 stability. **A** Acetylated TALDO1 K7 was identified by LC-MS/MS. **B** The sequences around TALDO1 K7 from different species were aligned. **C** Indicated plasmids were transfected into 293T cells, the acetylation was analyzed by IP and western blot. **D** Indicated plasmids were transfected into NPC cells, western blot analysis of TALDO1. **E** After NPC shHDAC6 stable cells were treated with CHX (0.1 μM), TALDO1 protein was analyzed by western blot. The amount of TALDO1 protein was quantitated and calculated by ImageJ software. **F** HK1 and SUNE1 cells were treated with MG132 (10 μM) or chloroquine (CHQ) (50 μM) for 12 h, western blot analysis was used to detect TALDO1 expression. **G** 293T cells were transfected with HA-Ub together with Vec or Flag-TALDO1. Anti-HA was used for IP and western blot was performed with Flag antibody to detect TALDO1 ubiquitination. **H** Indicated plasmids were transfected into 293T cells, the ubiquitination was analyzed by IP with a Flag antibody, followed by western blot for anti-HA. **I** Indicated plasmids were transfected into 293T cells, the ubiquitination was detected. **J** Flag-TALDO1 WT, K7R, or K7Q were transfected into 293T cells, the ubiquitination was examined. **K** 293T cells were transfected with Flag-TALDO1 together with HA-Ub, HA-K48R, or HA-K63R. The ubiquitination was detected.

Previous acetylation quantitative proteomic data showed a 5.7-fold increase in K7 acetylation of TALDO1 in LBH589-treated cells compared to controls (Fig. 5A), and K7 is highly conserved across species, indicating its significance as the main acetylation site (Fig. 5B). To verify this, Flag-TALDO1 K7R (mimicking deacetylation) and K7Q (mimicking acetylation) [26] mutants were transfected into 293T or NPC cells. The K7R mutant exhibited reduced TALDO1 acetylation compared to wild-type (WT) TALDO1 (Fig. 5C, D). To analyze whether acetylation is involved in the regulation of protein expression, cycloheximide assays in NPC cells showed that HDAC6 inhibition increased TALDO1 protein half-life (Figs. 5E, S7A), demonstrating that acetylation enhances TALDO1 stability. Further, treatment with the proteasome inhibitor MG132 or autophagy inhibitor chloroquine did not alter TALDO1 protein levels (Figs. 5F, S7B), indicating that acetylation did not affect the degradation of TALDO1 through ubiquitination or autophagy. Given that K48-linked ubiquitination leads to protein degradation, and K63-linked ubiquitination is predominantly associated with protein stability and signal transduction [27]. Therefore, we analyzed whether K63-linked ubiquitination is involved in the stability regulation of TALDO1. IP experiments confirmed the existence of ubiquitination modification in TALDO1 (Fig. 5G). HDAC6 knockdown, LBH589 treatment and p300 overexpression increased TALDO1 ubiquitination (Figs. 5H, I, S7C). Consistent with this, TALDO1 K7R showed lower ubiquitination than TALDO1 WT (Fig. 5J). Furthermore, IP experiments with ubiquitin plasmids (K48 and K63) and their mutants (K48R and K63R) revealed K63-linked ubiquitination as the major type for TALDO1 (Fig. S8A, 5K), and endogenous IP experiments also confirmed this finding (Fig. S8B). Thus, K7 deacetylation attenuates TALDO1 stability through inhibiting K63-linked ubiquitination. Moreover, biological function assays showed that the TALDO1 K7 mutation inhibited NPC cell proliferation and invasion (Fig. S9).

### Deacetylation inhibits SMURF1-mediated K63-linked ubiquitination of TALDO1

**Fig. 6:**
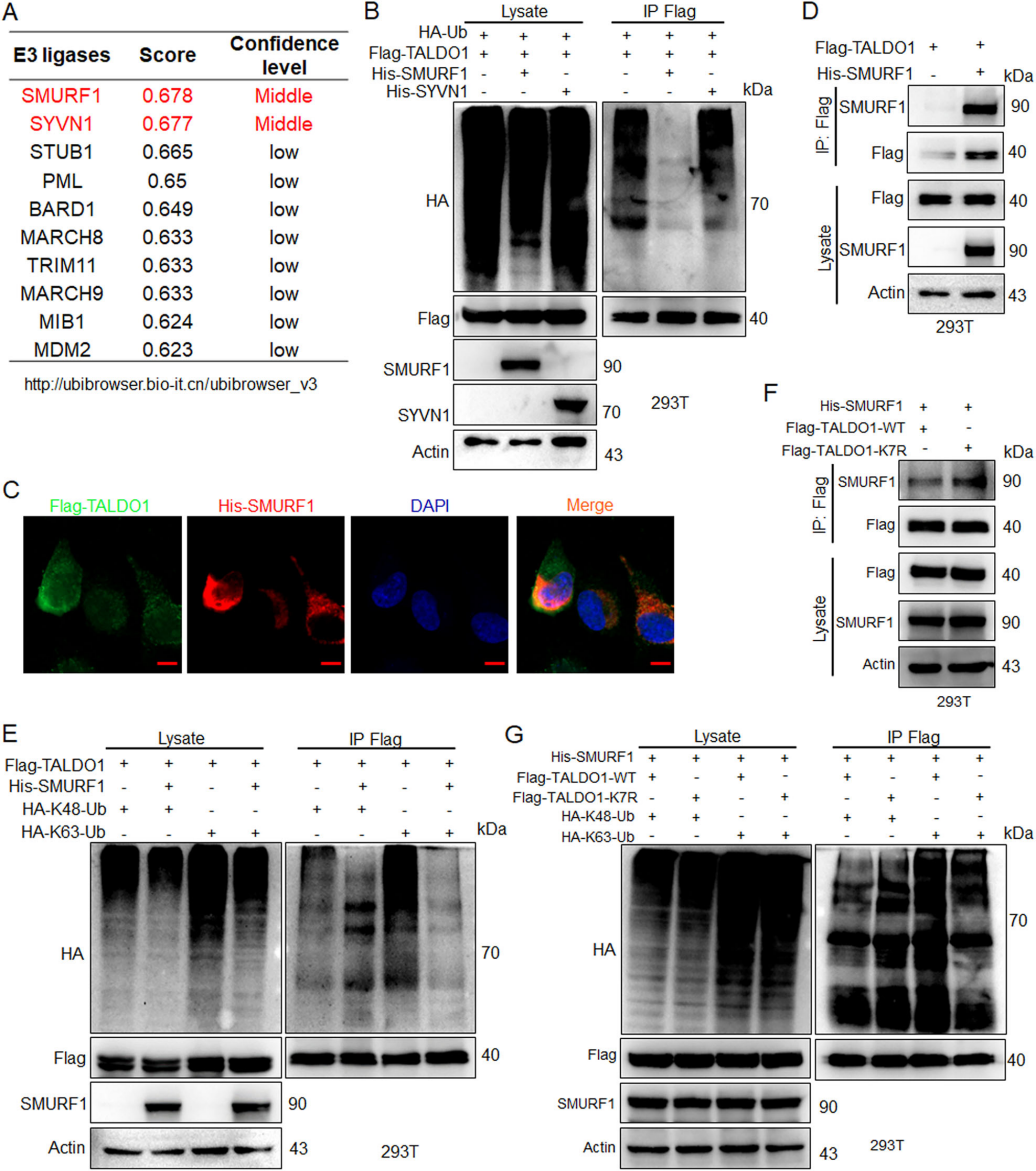
Deacetylation inhibits SMURF1-mediated K63-linked ubiquitination of TALDO1. **A** The E3 ligases associated with TALDO1 were screened using the UbiBrowser database (http://ubibrowser.ncpsb.org/), and ranked them by binding score and confidence level. **B** Indicated plasmids were transfected into 293T cells, the ubiquitination was examined. **C** After co-transfected Flag-TALDO1 and His-SMURF1 plasmids in HK1 cells, the colocalization of TALDO1 and SMURF1 was investigated by confocal microscopy. Scale bar, 5 μm. **D** After transfected Flag-TALDO1 and His-SMURF1 in 293T cells, interaction between TALDO1 and SMURF1 was detected by IP and western blot. **E** Indicated plasmids were transfected into 293T cells, the ubiquitination was detected. **F** His-SMURF1 and Flag-TALDO1-WT or K7R were transfected into 293T cells, interaction between TALDO1 and SMURF1 was examined by IP and western blot. **G** Indicated plasmids were transfected into 293T cells, the ubiquitination was detected.

To identify the E3 ligase for TALDO1, potential candidates were screened and ranked using the UbiBrowser database (Fig. 6A). IP experiments showed that SMURF1 overexpression decreased TALDO1 ubiquitination, while SYVN1 had no effect (Fig. 6B). Further IF and IP assays confirmed an interaction between SMURF1 and TALDO1 (Fig. 6C, D), and SMURF1 could significantly inhibit K63-linked ubiquitination and slightly promote K48-linked ubiquitination of TALDO1 (Fig. 6E). In addition, IP experiments revealed that the K7R mutation enhanced the interaction between TALDO1 and SMURF1 (Fig. 6F) and inhibited SMURF1-mediated K63-linked ubiquitination while promoting K48-linked ubiquitination (Fig. 6G). These results suggest that K7 deacetylation reduces SMURF1-mediated K63-linked ubiquitination and increases K48-linked ubiquitination of TALDO1, thereby decreasing TALDO1 stability.

### K7 deacetylation of TALDO1 promotes glycolysis in NPC cells independent of enzyme activity

**Fig. 7:**
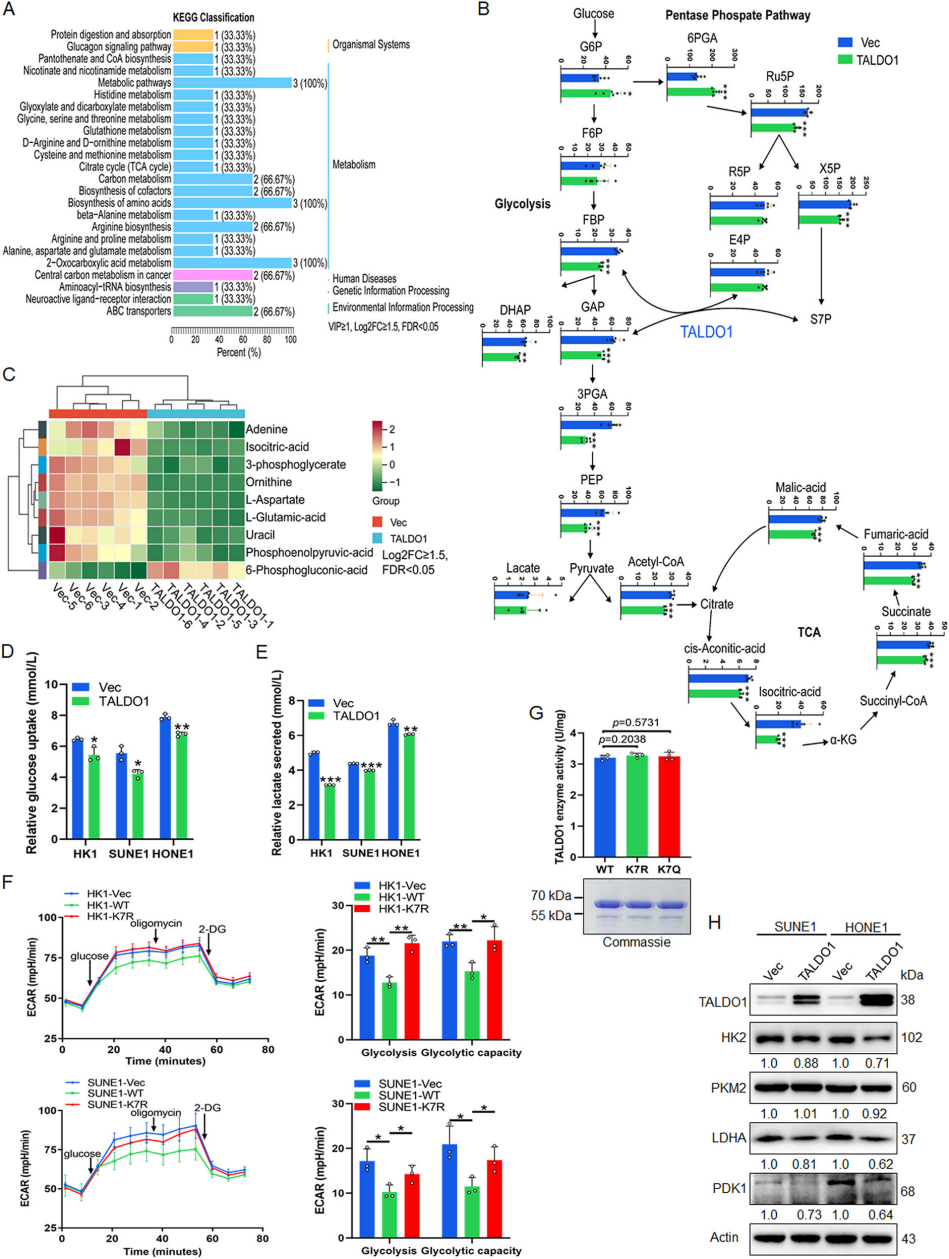
K7 deacetylation of TALDO1 promotes glycolysis in NPC cells independent of enzyme activity. **A**–**C** Targeted metabolomics with TALDO1-overexpressing and control SUNE1 cells was performed. **A** Differentially enriched pathways of SUNE1 cell with overexpressed TALDO1. Differential metabolites were identified based on Log2FC (fold change) ≥1.5, VIP ≥ 1, and FDR-adjusted *p* < 0.05. VIP values were extracted from the OPLS-DA results. **B** Altered metabolite levels in the main energy fluxes in TALDO1-overexpressing cells compared to the control cells. **C** Heatmap of most significant changed metabolites by the targeted metabolomics analysis. The differential metabolites were determined by Log2FC ≥ 1.5 and FDR-adjusted *p* < 0.05. **D**, **E** After transfection with pcDNA3.1-TALDO1 plasmids for 48 h, glucose uptake (**D**) and lactate production (**E**) in cell culture medium were measured using the glucose or lactate colorimetric assay kit. **F** After transfection with Vec, pcDNA3.1-TALDO1(WT), pcDNA3.1-TALDO1(K7R) plasmids into HK1 and SUNE1 cells, the extracellular acidification rate (ECAR) was measured using Seahorse XF assay Kit. The glycolysis and glycolysis capacity were analyzed. **G** Wild type, K7R, K7Q acetylated mutant recombinant proteins were used to detect TALDO1 enzyme activity in vitro. **H** Western blot assay was performed to assess the glycolysis rate-limiting enzyme levels in TALDO1 overexpression NPC cells. Data were shown as the mean ± SD of at least three independent experiments. *p < 0.05 and **p < 0.01 and ***p < 0.001.

TALDO1 is an important metabolic enzyme for the balance of metabolites in the PPP and glycolysis pathways [28]. To assess how TALDO1 affects the energy metabolism of NPC cells, targeted metabolomics analysis was performed on TALDO1-overexpressing NPC cells. Principal Component Analysis (PCA) showed clear separation between groups (Fig. S10A). TALDO1 overexpression led to increased upstream substrates such as Glucose-6-phosphate (G6P) and 6-phosphogluconic acid (6PGA) in glucose metabolism, but resulted in a decrease in downstream glycolysis and TCA cycle substrates, notably 3-phosphoglycerate (3PGA), phosphoenolpyruvate (PEP) and isocitric acid. However, the changes in its direct substrates such as erythrose-4-pyosphate (E4P) were not significant (Figs. 7A–C, S10B). These results suggest that TALDO1 may reduce glycolysis and TCA cycle in NPC cells. Further, glucose uptake and lactate production assays demonstrated that TALDO1 inhibited both processes (Fig. 7D, E). Meanwhile, Seahorse assays showed that TALDO1 overexpression decreased glycolysis and glycolytic capacity in NPC cells, and this effect was reversed by the K7R mutant (Fig. 7F). Consistent with this, the glycolysis and glycolytic capacity were also reduced in shHDAC6 NPC cells (Fig. S10C). However, enzyme activity assays indicated K7 acetylation did not significantly affect TALDO1 activity (Fig. 7G), suggesting that TALDO1 may influence glucose metabolism independent of its enzymatic activity but rather through moonlighting functions. Furthermore, we assessed the expression of key rate-limiting enzymes in the glycolysis and TCA cycle. Western blot analysis showed that the glycolytic rate-limiting enzymes HK2 and LDHA, as well as the TCA cycle enzyme PDK1, were downregulated in TALDO1-overexpressing cells (Fig. 7H). Taken together, these results demonstrate that deacetylation of TALDO1 enhances glycolysis of NPC cells independently of its enzymatic activity.

Additionally, we aimed to clarify whether TALDO1-mediated glycolysis inhibition is involved in the proliferation and invasion of NPC cells. CCK-8, colony formation and Transwell assay results showed that stable knockdown of TALDO1 significantly promoted the proliferation and invasion of NPC cells, and further treatment with 2-DG (competitive glycolysis inhibitor) reversed the effect (Fig. S11A–D). Further, glycolysis enzyme expression was significantly increased in the TALDO1 knockdown group, but this increase was mitigated by 2-DG treatment (Fig. S11E). Collectively, these results demonstrate that TALDO1 inhibits the proliferation and invasion of NPC cells by suppressing glycolysis.

### Deacetylation inhibits nuclear translocation of TALDO1 and promotes glycolysis of NPC cells

**Fig. 8:**
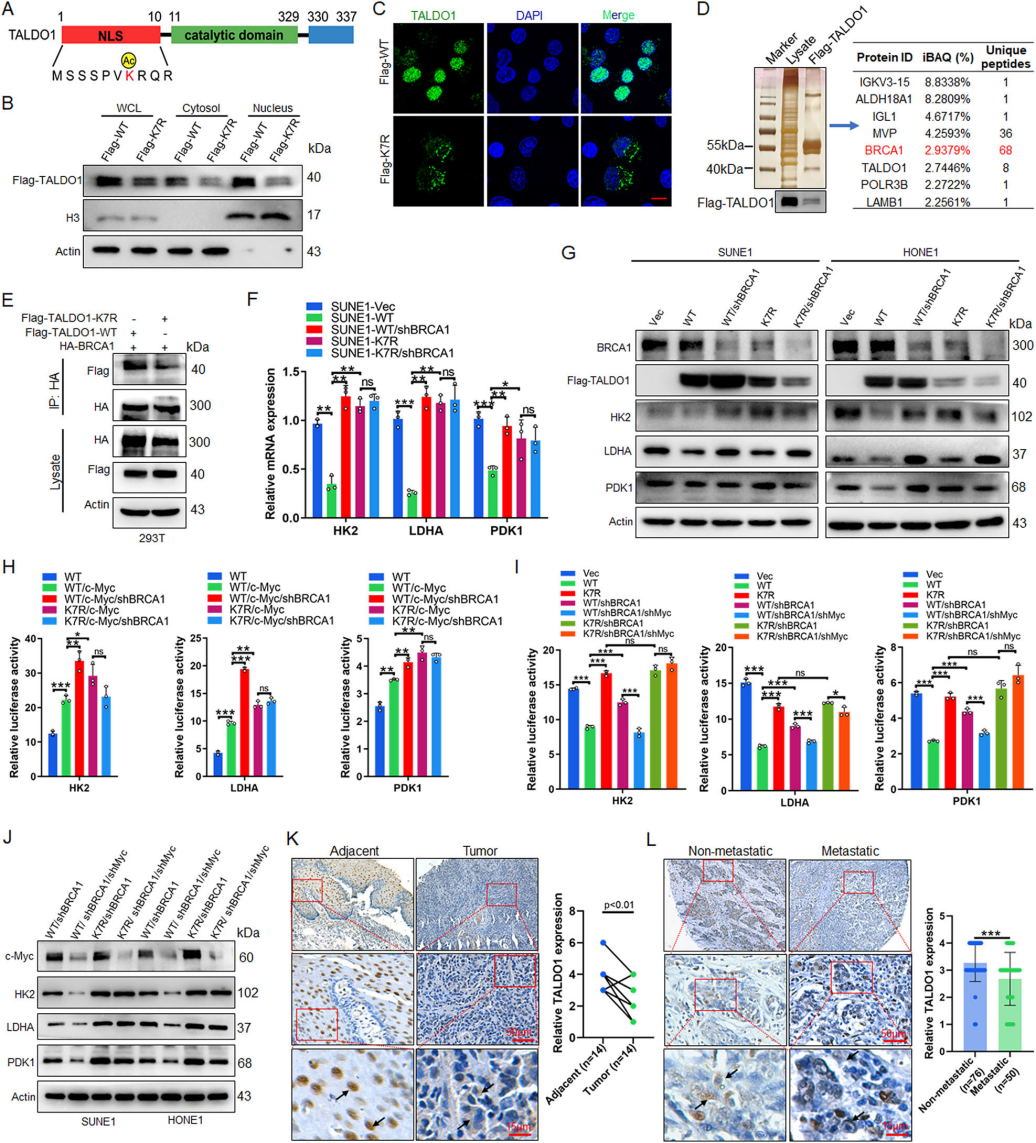
Deacetylation inhibits nuclear translocation of TALDO1 and promotes glycolysis of NPC cells. **A** Schematic diagram of the domain of TALDO1 protein. **B** After transfecting SUNE1 cells with Flag-TALDO1-WT or Flag-TALDO1-K7R plasmids for 48 h, cytoplasmic and nuclear fractions were obtained using the nuclear cytoplasmic extraction reagent kit, TALDO1 distribution in the cytoplasm and nucleus was analyzed by western blot, with H3 as the nuclear marker and actin as the cytoplasmic marker. **C** The subcellular localization of TALDO1 was analyzed by IF assay 48 h after transfection of the indicated plasmid in HK1 cells. **D** 48 h after HK1 cells transfected Flag-TALDO1 plasmid, the proteins interacting with TALDO1 were analyzed by protein mass spectrometry. **E** HA-BRCA1 and Flag-TALDO1-WT or K7R were transfected into 293T cells, interaction between TALDO1 and BRCA1 was examined by IP and western blot. **F** The mRNA expression of HK2, LDHA and PDK1 was detected by qPCR after indicated plasmid was transfected into SUNE1 cells for 48 h. **G** The protein expression of HK2、LDHA and PDK1 was detected by western blot after indicated plasmid was transfected into SUNE1 and HONE1 cells for 48 h. **H**, **I** After the indicated plasmids were transfected into SUNE1 cells for 48 h, the transcriptional activities of HK2, LDHA and PDK1 were detected by Dual luciferase reporter assay. **J** After the indicated plasmids were transfected into SUNE1 and HONE1 cells for 48 h, the protein expression of c-Myc, HK2, LDHA and PDK1 was detected by western blot. **K**, **L** The expression and subcellular localization of TALDO1 were assessed by immunohistochemistry in NPC and adjacent nasopharyngeal mucosa/epithelial tissues (**K**), as well as in metastatic and non-metastatic NPC tissues (**L**). Data were shown as the mean ± SD of at least three independent experiments. *p < 0.05 and **p < 0.01 and ***p < 0.001.

Traditionally, metabolic reprogramming was linked to enzyme activity and substrate concentrations. Recent findings suggest that modulating enzyme moonlighting functions also enables tumor cells to adapt during tumorigenesis and progression [29, 30]. The two TALDO1 isoforms, generated by alternative translation initiation, show distinct nuclear and cytoplasmic localization. The long isoform is crucial for nuclear targeting, but the mechanisms regulating its nuclear localization and its metabolic role remain unclear [31]. We analyzed the protein domains of TALDO1 and found that its acetylation site, K7, is located within the nuclear localization signal (NLS) (Fig. 8A). IF and nuclear-cytoplasmic fractionation experiments showed that wild-type TALDO1 primarily localizes in the nucleus, while the K7R mutant predominantly localizes in the cytoplasm (Fig. 8B, C), indicating that deacetylation inhibits TALDO1 nuclear translocation. To explore the mechanism by which nuclear TALDO1 exerts its tumor-suppressive function, we performed mass spectrometry to analyze TALDO1-binding proteins in NPC cells. The results suggested that the tumor suppressor protein BRCA1, known for its transcriptional regulatory properties, might interact with TALDO1 (Fig. 8D). IP experiments confirmed this, and K7R significantly weakened the TALDO1-BRCA1 interaction (Fig. 8E). Previous studies have demonstrated that BRCA1 can directly inhibit the transcriptional activity of c-Myc [32], thereby reducing the expression of glycolysis-related metabolic enzymes such as HK2, LDHA, and PDK1 [33, 34]. We knocked down BRCA1 expression in SUNE1 cells overexpressing either wild-type or K7R mutant TALDO1. The results showed that wild-type TALDO1 overexpression decreased the mRNA and protein expression of HK2, LDHA, and PDK1, while the K7R mutation reversed this effect. Furthermore, BRCA1 knockdown alleviated the decrease in metabolic enzyme mRNA and protein expression caused by TALDO1 overexpression, but had no significant effect in the K7R group (Fig. 8F, G). We also constructed reporter plasmids for HK2, LDHA, and PDK1, and dual-luciferase assays demonstrated that wild-type TALDO1 overexpression inhibited the transcriptional activity of these genes, and this effect was reversed by the K7R mutation. BRCA1 knockdown counteracted the changes in transcriptional activity induced by TALDO1 overexpression, whereas knocking down c-Myc expression negated the effect of BRCA1, with no significant changes observed in the K7R group (Fig. 8H, I). Western blot experiments further confirmed these findings (Fig. 8J). These results suggest that acetylation-mediated nuclear translocation of TALDO1 inhibits glycolysis by interacting with BRCA1, which suppresses c-Myc-mediated transcriptional activation of glycolytic enzymes. IHC staining of NPC tissue microarrays revealed that TALDO1 expression was lower in tumor tissues compared to paired adjacent tissues. Moreover, primary tumors with metastases exhibited lower TALDO1 expression than those without metastases. Notably, as the malignancy of the tissue increased, nuclear localization of TALDO1 decreased significantly (Fig. 8K, L). Taken together, these results indicate that inhibition of glycolysis by TALDO1 is dependent on acetylation-mediated nuclear translocation.

### Deacetylation disrupts nuclear translocation of TALDO1 to promote NPC proliferation and migration via the BRCA1/c-Myc axis

**Fig. 9:**
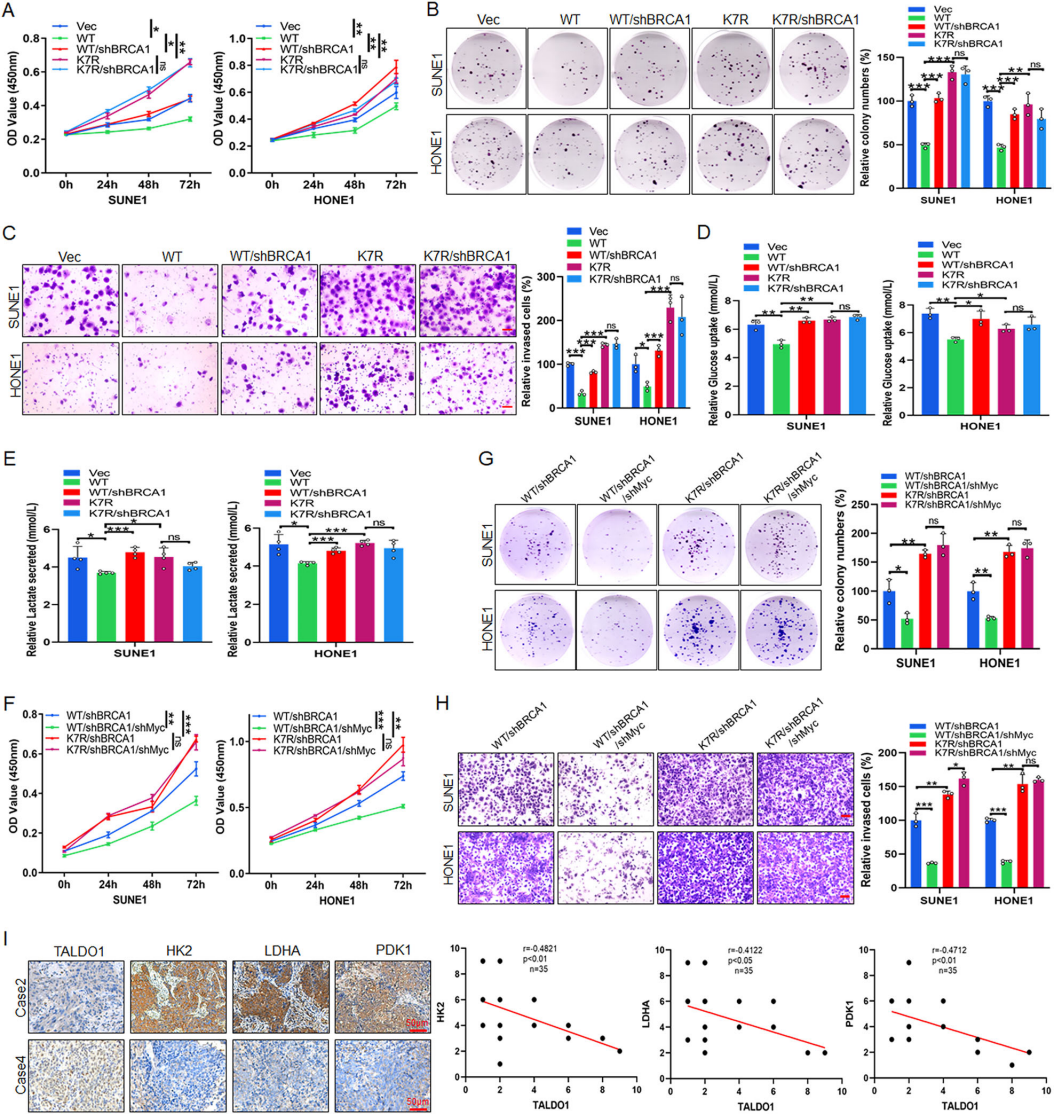
Deacetylation-mediated nuclear translocation of TALDO1 promotes NPC proliferation and migration via the BRCA1/c-Myc axis. **A**–**E** After transfections of the indicated plasmids into NPC cells for 48 h. **A**, **B** CCK-8 assay and colony forming assay were performed to examine the cell proliferation. **C** Transwell assays were carried out to detect cell invasion. Scale bar, 50 μm. **D**, **E** After transfection with indicated plasmids for 48 h, glucose uptake (**D**) and lactate production (**E**) in cell culture medium were measured using the glucose or lactate colorimetric assay kit. **F**–**H** After transfection with indicated plasmids for 48 h, **F**, **G** CCK-8 assay and colony forming assay were performed to examine the cell proliferation. **H** Transwell assays were carried out to detect cell invasion. Scale bar, 50 μm. **I** The expression and correlation of TADLO1 and HK2, LDHA or PDK1 were analyzed by IHC staining of clinical NPC tissues. Sample size (*n* = 35), Pearson correlation coefficient (r) and *p*-value (two-tailed) are indicated. Data were shown as the mean ± SD of at least three independent experiments. No significant (ns), *p < 0.05 and **p < 0.01 and ***p < 0.001.

To investigate the role of the BRCA1/c-Myc axis in TALDO1-mediated glycometabolic inhibition of NPC cells, biological functional assays revealed that BRCA1 knockdown in wild-type TALDO1-overexpressing NPC cells reversed the inhibition of cell proliferation and migration induced by TALDO1 overexpression. However, this effect was absent in the K7R mutant group (Fig. 9A–C). Consistently, glucose uptake and lactate production assays demonstrated that TALDO1 inhibits glycolysis in NPC cells via BRCA1, a loss of function in the K7R deacetylation mutant (Fig. 9D, E). Further biological functional assays showed that c-Myc knockdown significantly suppressed the cell proliferation and migration promotion effects caused by BRCA1 downregulation in wild-type TALDO1-overexpressing NPC cells. This suppression was not observed in the K7R mutant group (Fig. 9F–H). Additionally, IHC staining of NPC tissues revealed a significant negative correlation between TALDO1 expression and glycolysis-related enzymes HK2, LDHA, and PDK1 (Fig. 9I). These results suggest that deacetylation-mediated disruption of TALDO1 nuclear translocation promotes glycolysis through the BRCA1/c-Myc axis, thereby enhancing NPC cell proliferation and migration.

## Discussion

Several studies have revealed the essential role of TALDO1 in tumor progression. The co-expression of SLC1A5 and TALDO1 in estrogen receptor-positive breast cancer was associated with poor clinical outcomes in patients treated with endocrine therapy [35]. TALDO1 has also been associated with drug resistance in HER2-positive breast cancer, and deletion of TALDO1 and HER2 inhibition improved HER2-positive breast cancer treatment [36]. Higher TALDO1 expression in upper tract urothelial carcinoma could predict poor prognosis [37]. In contrast, TALDO1 knockout mice spontaneously develop hepatocellular carcinoma (HCC), suggesting that TALDO1 can inhibit HCC carcinogenesis [38]. Consistently, the TALDO1 deletion mutant phenotype in humans may lead to early HCC formation [39]. Increased TALDO1 expression in HNSC predicted better recurrence-free survival [40]. These results imply that TALDO1 may have a dual role in tumors, and that it acts as an oncogene or as a tumor suppressor depending on the tumor type. In this study, we found that TALDO1 expression was downregulated in NPC and associated with poor prognosis. Ectopic expression of TALDO1 significantly suppressed NPC proliferation and metastasis in vitro and in vivo. Our findings demonstrate that TALDO1 functions as a tumor suppressor in NPC.

Post-translational modifications are key regulators of protein expression [41]. Here, we demonstrate for the first time that the deacetylase HDAC6 interacts with TALDO1, mediating its deacetylation and reducing its protein expression, while the acetyltransferase p300 enhances TALDO1 acetylation, increasing its protein expression. Meanwhile, we found that K7 deacetylation inhibits E3 ligase SMURF1-mediated K63-linked ubiquitination and promotes K48-linked ubiquitin-mediated degradation of TALDO1, thereby reducing its protein stability. Our study elucidated the regulatory mechanism of TALDO1 expression from the perspective of the interaction between protein acetylation and ubiquitination.

TALDO1 is the rate-limiting enzyme of PPP that converts the glycolytic intermediate GAP to F6P and plays an important role in linking glycolysis and PPP [42]. Our metabolic analysis demonstrated that TALDO1 overexpression significantly reduced glycolysis and TCA metabolism. Meanwhile, we observed that the TALDO1 K7R deacetylation mutant enhanced the glycolytic capacity of NPC cells. However, in vitro enzyme activity assays demonstrated that the TALDO1 K7R deacetylation mutant did not alter its enzymatic activity. It is suggested that deacetylation of TALDO1 may play a role in promoting the glycolysis of tumor cells through moonlighting functions.

In recent years, it has been found that the metabolism reprogramming can be realized not only by regulating the classical function of metabolic enzyme activity, but also by regulating its moonlighting function to give tumor cells adapt to different demands in the development of tumor [5, 43]. Research has found that the main modes of the moonlighting function action of metabolic enzymes in tumors includes regulating gene transcription: direct interaction with and modulation of transcription factors and transcriptional co-regulators, even some enzymes may directly serve as transcription factors or regulators after nuclear translocation, whereas subcellular localization of some other enzymes may regulate gene transcription through histone modification; The intracellular re-distribution of some enzymes may establish novel protein interactions, leading to changes in the functionality of signaling pathways; And multiple studies revealed the direct regulation of protein substrates by metabolic enzymes with kinase activity, etc [44, 45]. For example, ERK2 mediated phosphorylation of PKM2 at Ser37, resulting in PKM2 binding to importin α5 and undergoing nuclear translocation. Nuclear PKM2 functions as a coactivator of β-catenin, inducing c-Myc expression, which led to the upregulation of GLUT1 and LDHA, thereby enhancing glycolysis and promoting glioblastoma progression [46]. NAT10 stabilizes ACLY via K468 acetylation, thereby increasing nuclear acetyl-CoA levels, which upregulate H3K27ac-mediated transcription of CYP2C9 and PIK3R1, consequently driving chemoresistance in HCC [47]. Additionally, the elevated expression of enolase-1 (ENO1) facilitated its interaction with choline kinase α (CHKα), which competitively disrupted the CHKα-TRIM25 binding, increased the stability of CHKα, thereby enhancing choline metabolism and accelerating brain tumor growth [48]. Moreover, Creatine kinase B (CKB) phosphorylates GPX4 at S104, functioning as a protein kinase, which suppresses HSC70-mediated autophagic degradation of GPX4, thereby attenuating ferroptosis and advancing HCC progression [49].

The TALDO1 gene produces two isoforms: a full-length 337-amino acid protein and a shorter version missing the first 10 amino acids, which lacks nuclear localization. While subcellular localization of TALDO1 is not crucial for its activity in the PPP, it influences NIH/3T3 cell metabolism, including the TCA cycle, nucleotide metabolism, and glycolysis [31]. In this study, unlike nuclear-translocating metabolic enzymes such as PKM2 and ACLY that directly act as transcriptional coactivators or remodel histone acetylation, nuclear-translocated TALDO1 suppresses the c-Myc transcriptional activation through binding to the transcriptional corepressor BRCA1. Consistently, recent studies demonstrate that nuclear-localized glutamate-cysteine ligase modifier subunit (GCLM) competitively interacts with the NF-κB repressing factor (NKRF), thereby promoting NF-κB activity and augmenting chemoresistance in colorectal cancer [50].

**Fig. 10:**
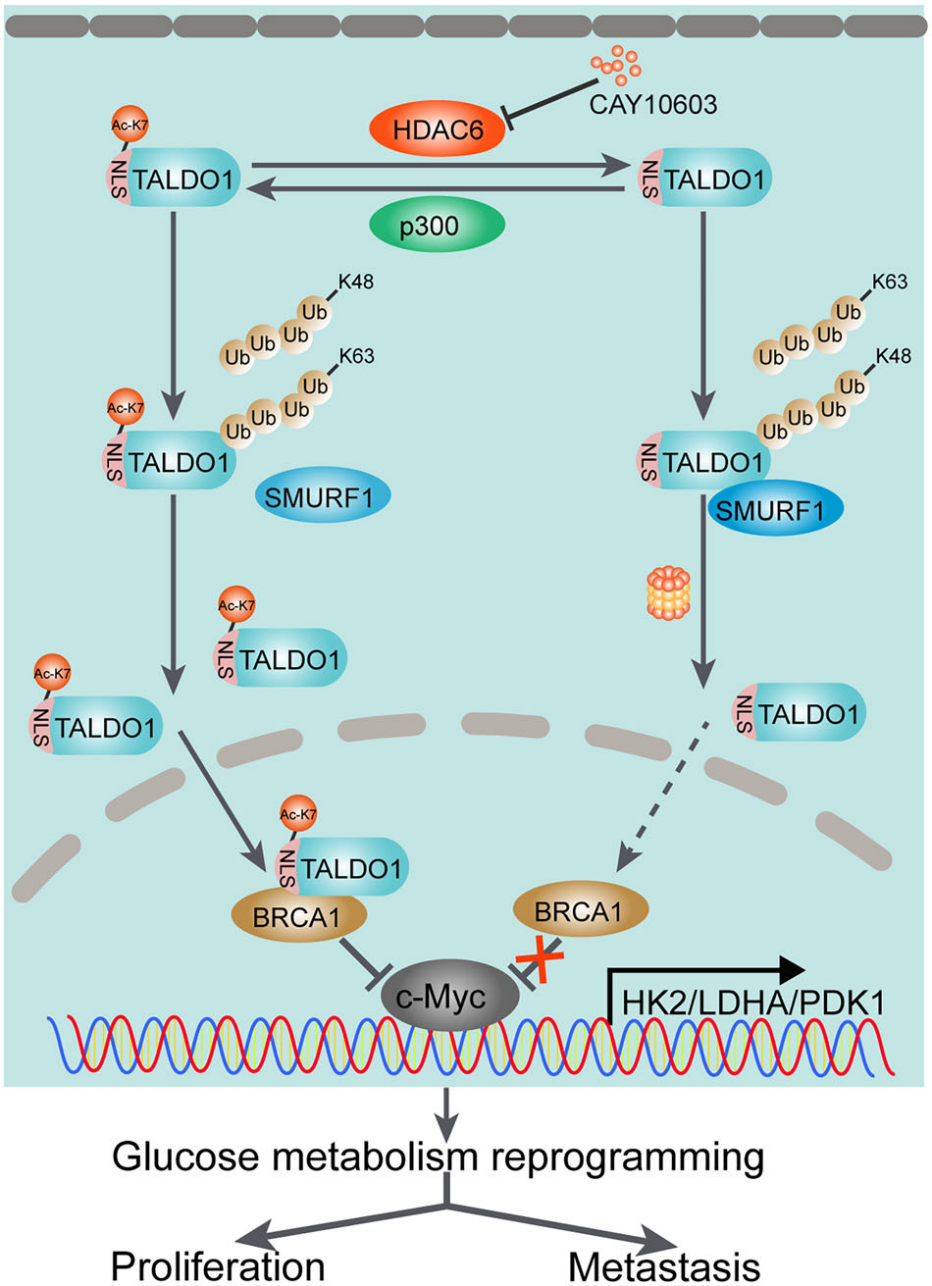
Graphical model. Schematic diagram illustrates that the potential mechanisms by which deacetylation-dependent regulation of TADLO1 stability and nuclear translocation promotes glycolysis and malignant progression of NPC.

In conclusion, our study highlighted the critical regulatory role of deacetylation of TALDO1 in regulating its stability and nuclear translocation, as well as its impact on glycolysis. Meaningfully, we found that nuclear TALDO1 possessed a moonlighting function, which interrupted c-Myc-mediated transcriptional activation of HK2/LDHA/PDK1 through binding to BRCA1 (Fig. 10). This study provides a novel biomarker and intervention strategy for NPC from the perspective of protein acetylation modification.

## Supplementary information

Supplementary Materials

original western-blots

## Data Availability

All datasets generated and analyzed during this study are included in this published article and its Supplementary material files. Additional data are available from the corresponding author upon reasonable request.
