# Supplementary material for: Deacetylation of TALDO1 by HDAC6 promotes glycolysis and nasopharyngeal carcinoma progression through a moonlighting function
Source: Cell Death Dis. 2025 Oct 21;16(1):743. doi: 10.1038/s41419-025-08057-2 (PMC12540763; doi:10.1038/s41419-025-08057-2)
Supplement: Supplementary file 1 — Supplementary Materials [file 41419_2025_8057_MOESM1_ESM.docx]

**Supplementary Materials**

**Deacetylation of TALDO1 by HDAC6 promotes glycolysis and nasopharyngeal carcinoma progression through a moonlighting function**

Xingzhi Peng, Peijun Zhou, Kun Zhang, Likang Chen, Min Tang, Qin Zhou, Jinwu Peng, Lifang Yang

**Content**

● **Fig. S1.** The TALDO1 mRNA level in HNSC.

● **Fig. S2.** The effect of TALDO1 overexpression plasmid in NPC cells.

● **Fig. S3.** HDAC6 is the main deacetylase of TALDO1.

● **Fig. S4.** P300 is an acetyltransferase of TALDO1.

● **Fig. S5.** HDAC6 promotes the proliferation and invasion of NPC cells.

● **Fig. S6.** HDAC6-specific inhibitor CAY10603 inhibits the proliferation and metastasis of NPC.

● **Fig. S7.** Deacetylation suppresses the TALDO1 stability.

● **Fig. S8.** The ubiquitination of TALDO1 is mainly K63 linked ubiquitination.

**● Fig. S9.** K7 deacetylation of TALDO1 promotes the proliferation and migration of NPC cells.

**● Fig. S10.** Significant changes in metabolite levels in TALDO1 overexpressing SUNE1 cells.

**● Figure S11.** TALDO1 inhibited the proliferation and invasion of NPC cells by weakening glycolysis.

● **Table S1.** Clinical characteristics of NPC patients (tissue microarrays).

● **Table S2.** Clinical data of NPC patients (serum samples).

● **Table S3.** Oligonucleotide sequences.

● **Table S4.** Acetylated metabolism enzymes in LBH589 treated HK1 cells.

**Supplementary Figures**

**
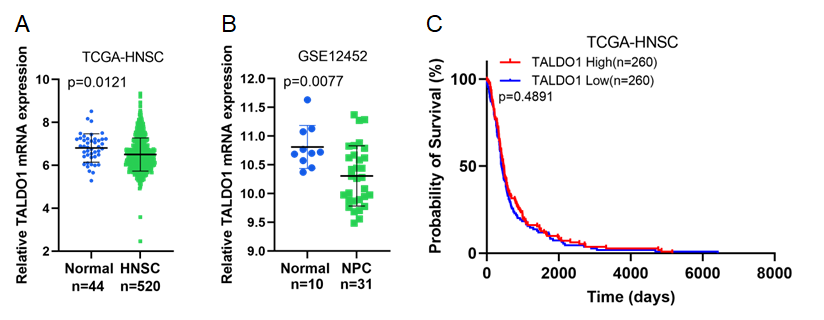
**

**Fig. S1. The TALDO1 mRNA level in HNSC.** **A-B** Analysis of TALDO1 mRNA levels using TCGA-HNSC **(A)** and GEO **(B)** datasets. **C** Overall survival rates in HNSC patients with low or high TALDO1 expression assessed by Kaplan–Meier analysis.

**
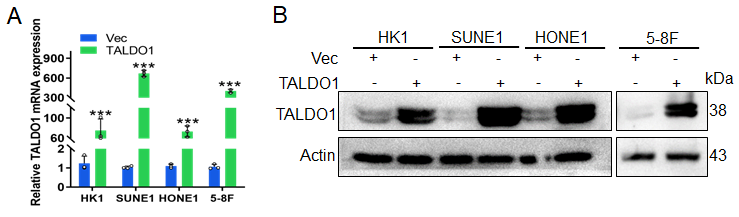
**

**Fig. S2.** **The effect of TALDO1 overexpression plasmid in NPC cells.** **A-B** After transfected pcDNA3.1-TALDO1 plasmids in HK1, SUNE1, HONE1 and 5-8F cells for 48 h, qPCR **(A)** and western blot **(B)** were performed to detect TALDO1 expression. Data were shown as the mean ± SD of at least three independent experiments. ***P<0.001.

**
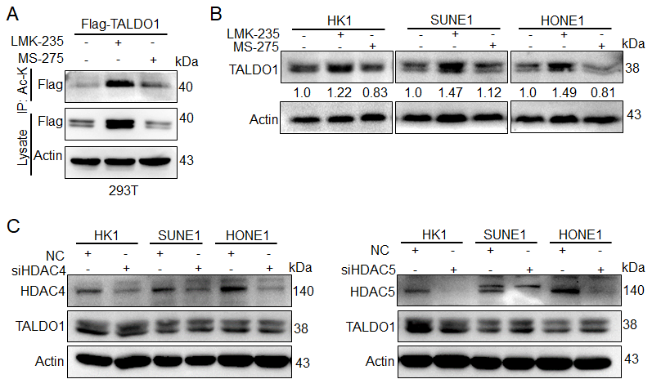
**

**Fig. S3.** **HDAC6 is the main deacetylase of TALDO1.** **A** After transfected Flag-TALDO1 in 293T cells and treated with selective HDAC inhibitors LMK-235 (0.5μM, 24 h) or MS-275 (1μM, 24 h), western blot analysis of exogenous acetylation levels of TALDO1 was performed by IP with an anti-acetylated lysine antibody. **B** Western blot analysis of TALDO1expression in NPC cells treated with LMK-235 or MS-275. **C** After transfected siHDAC4 or siHDAC5 in HK1, SUNE1 and HONE1 cells for 48 h, western blot analysis was performed to detect TALDO1 expression.

**
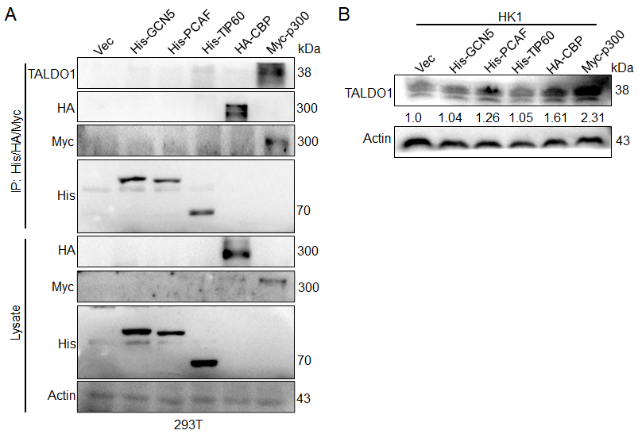
**

**Fig. S4.** **P300 is an** **acetyltransferase of TALDO1. A** Indicated plasmids were transfected into 293T cells for 48 h, and interaction between different acetyltransferase and TALDO1 was detected by IP and western blot. **B** After transfected different acetyltransferase plasmids in HK1 cells for 48 h, western blot was used to analyze of TALDO1 expression.

**
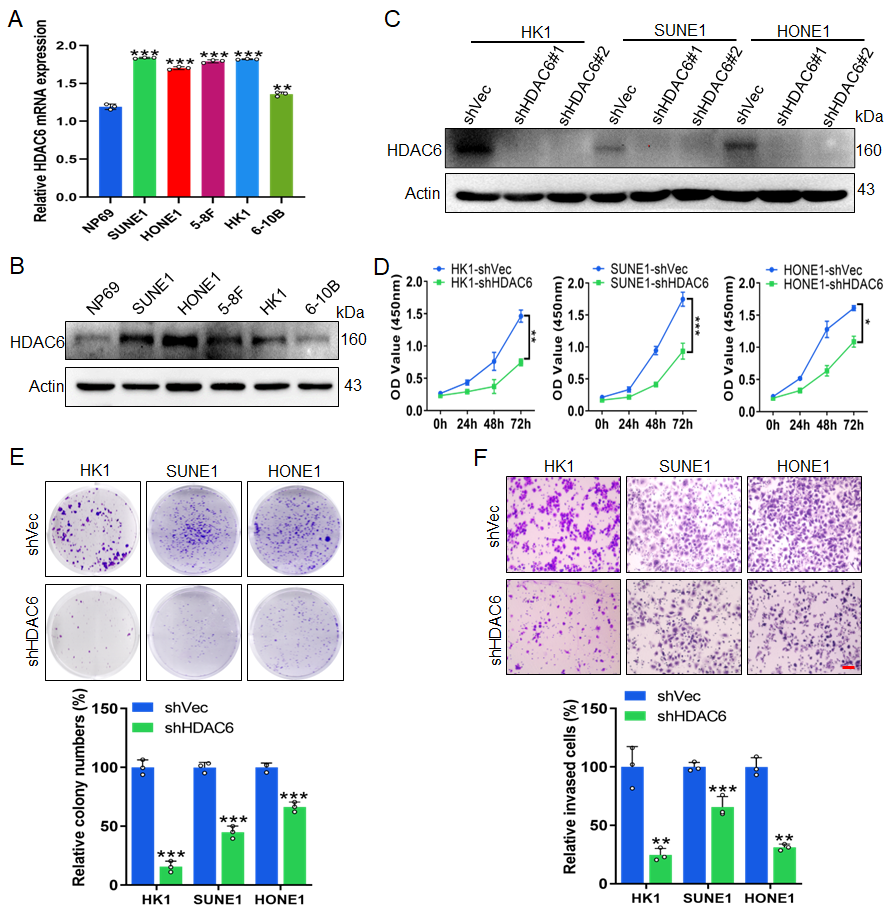
**

**Fig. S5.** **HDAC6** **promotes the proliferation and invasion of NPC cells**. **A-B** qPCR **(A)** and western blot **(B)** analysis of HDAC6 expression in five NPC cell lines (SUNE1, HONE1, 5-8F, 6-10B, and HK1) compared to normal nasopharyngeal mucosal/epithelial cell lines NP69. **C** Western blot analysis of HDAC6 expression in stable knockdown HDAC6 NPC cells. Used stable knockdown HDAC6 NPC cells, **D-E** CCK-8 assay **(D)** and colony forming assay **(E)** were carried out to examine the cell proliferation, **F** Transwell assays were performed to detect cell invasion. Scale bar, 50μm. Data were shown as the mean ± SD of at least three independent experiments. *P<0.05 and **P<0.01 and ***P<0.001.

**
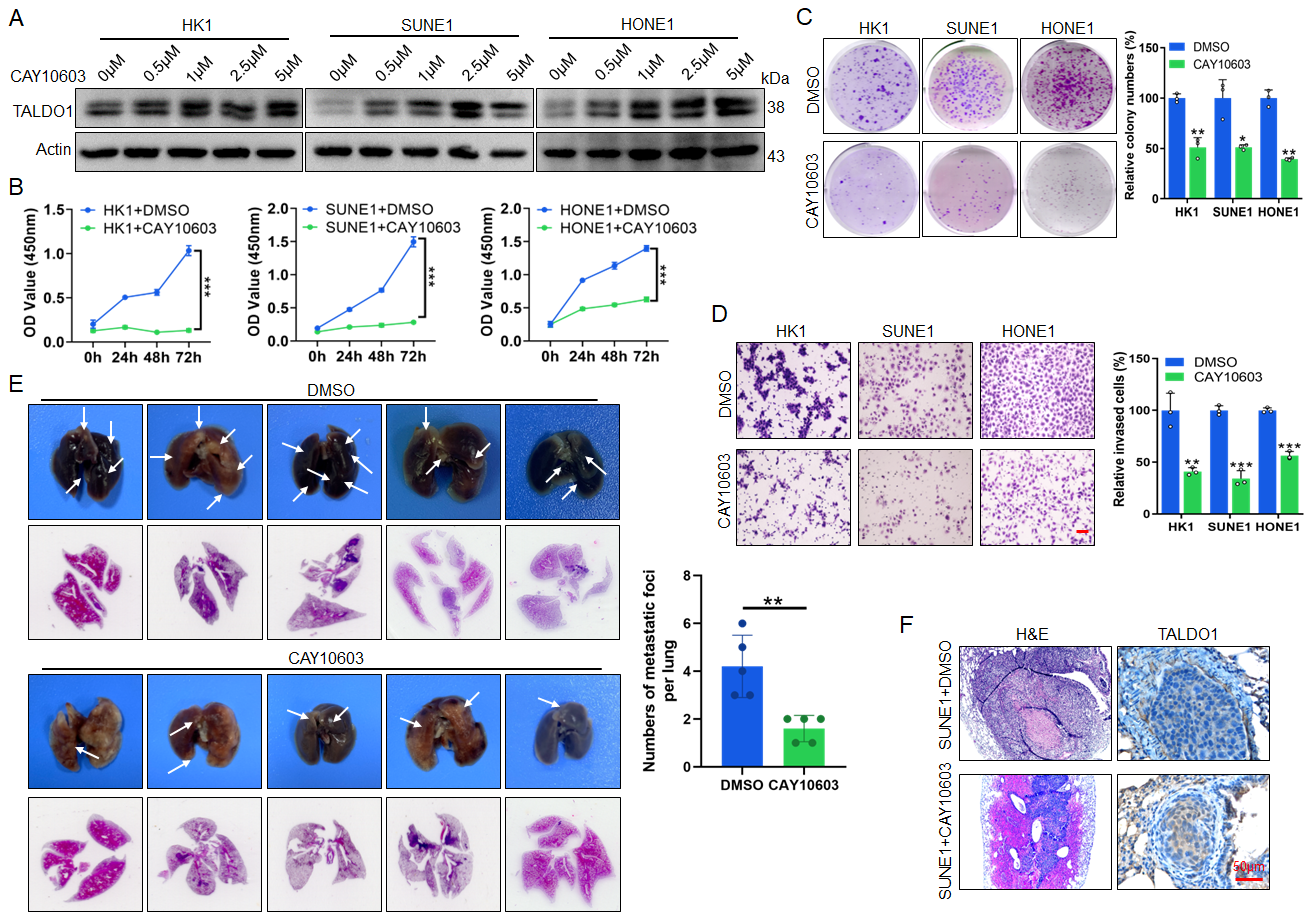
**

**Fig. S6.** **HDAC6-specific inhibitor CAY10603 inhibits the proliferation and metastasis of NPC.** **A** Western blot analysis was performed to detect TALDO1 levels in NPC cells after treatment with gradient concentration CAY10603 for 48 h. After NPC cells were treated with CAY10603 (1μM), **B-C** CCK-8 assay **(B)** and colony forming assay **(C)** were carried out to examine the cell proliferation, **D** Transwell assays were performed to detect cell invasion. Scale bar, 50μm. **E-F** After SUNE1 cells were injected into the lateral tail vein of 5-week-old female nude mice (n=5) and then treated with CAY10603 (20 mg/kg, intraperitoneal injection, every three days for one month), images and quantitative data of lung metastatic foci **(E)**, representative images of H&E staining of lung foci and IHC staining of HDAC6 and TALDO1 in lung foci were shown **(F)**. Scale bar, 50μm. Data were shown as the mean ± SD of at least three independent experiments. *P<0.05 and **P<0.01 and ***P<0.001.

**
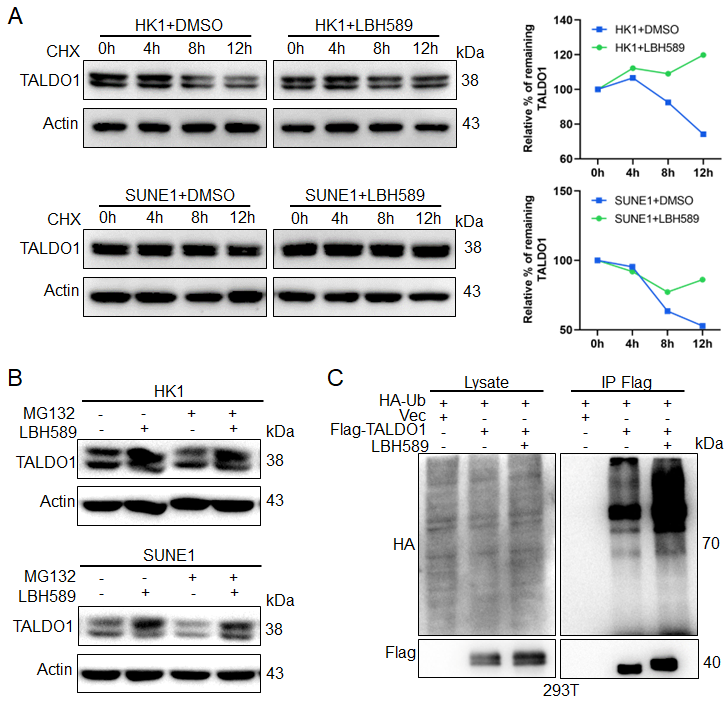
**

**Fig. S****7.** **Deacetylation suppresses the TALDO1 stability. A** HK1 and SUNE1 cells were cultured with LBH589 (100nM) for 24 h and then treated with the protein synthesis inhibitor cyclohexanone (CHX, 0.1μM), TALDO1 protein was analyzed by western blot. The amount of TALDO1 protein was quantitated and calculated by Image J software. **B** HK1 and SUNE1 cells were cultured with LBH589 and then treated with the proteasome inhibitor MG-132 (10μM) for 12 h. TALDO1 protein was analyzed by western blot. **C** Flag-TALDO1 and HA-Ub were transfected into 293T cells with or without LBH589 treatment, the ubiquitination levels were analyzed by IP with a Flag antibody followed by western blot for anti-HA.


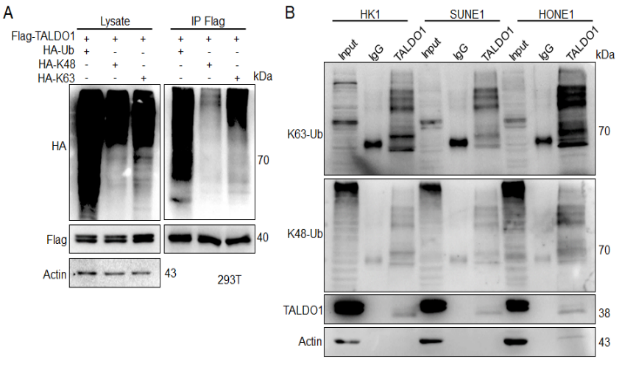


**Fig. S8. The ubiquitination of TALDO1 is mainly K63 linked ubiquitination. A** 293T cells were transfected with Flag-TALDO1 together with HA-Ub, HA-K48 or HA-K63, the ubiquitination levels were detected. **B** The K63- and K48-linked ubiquitination of endogenous TALDO1 were detected by IP with anti-TALDO1 followed by western blot using a K63- and K48-specific ubiquitination antibody.

**
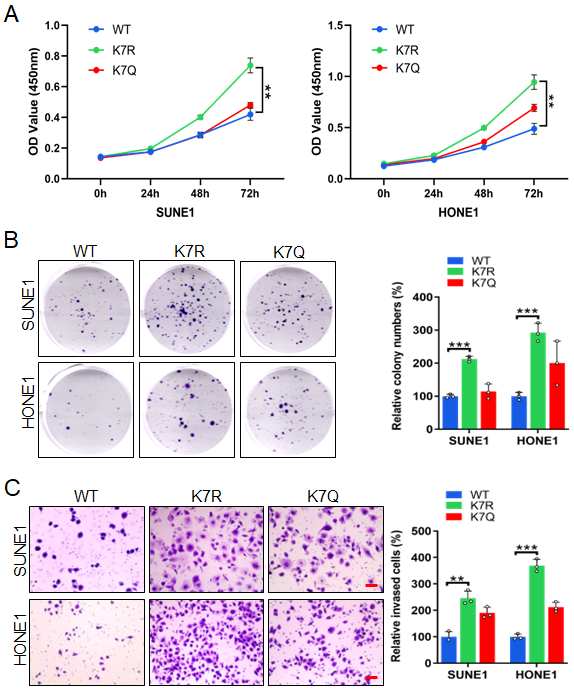
**

**Fig. S9. K7 deacetylation of TALDO1 promotes the proliferation and migration of NPC cells. A-C** After transfections of pcDNA3.1-TALDO1 plasmid into NPC cells for 48 h. **A-B** CCK-8 assay **(A)** and colony forming assay **(B)** were performed to examine the cell proliferation. **C** Transwell assays were carried out to detect cell invasion. Scale bar, 50μm. Data were shown as the mean ± SD of at least three independent experiments. **P<0.01 and ***P<0.001.

**
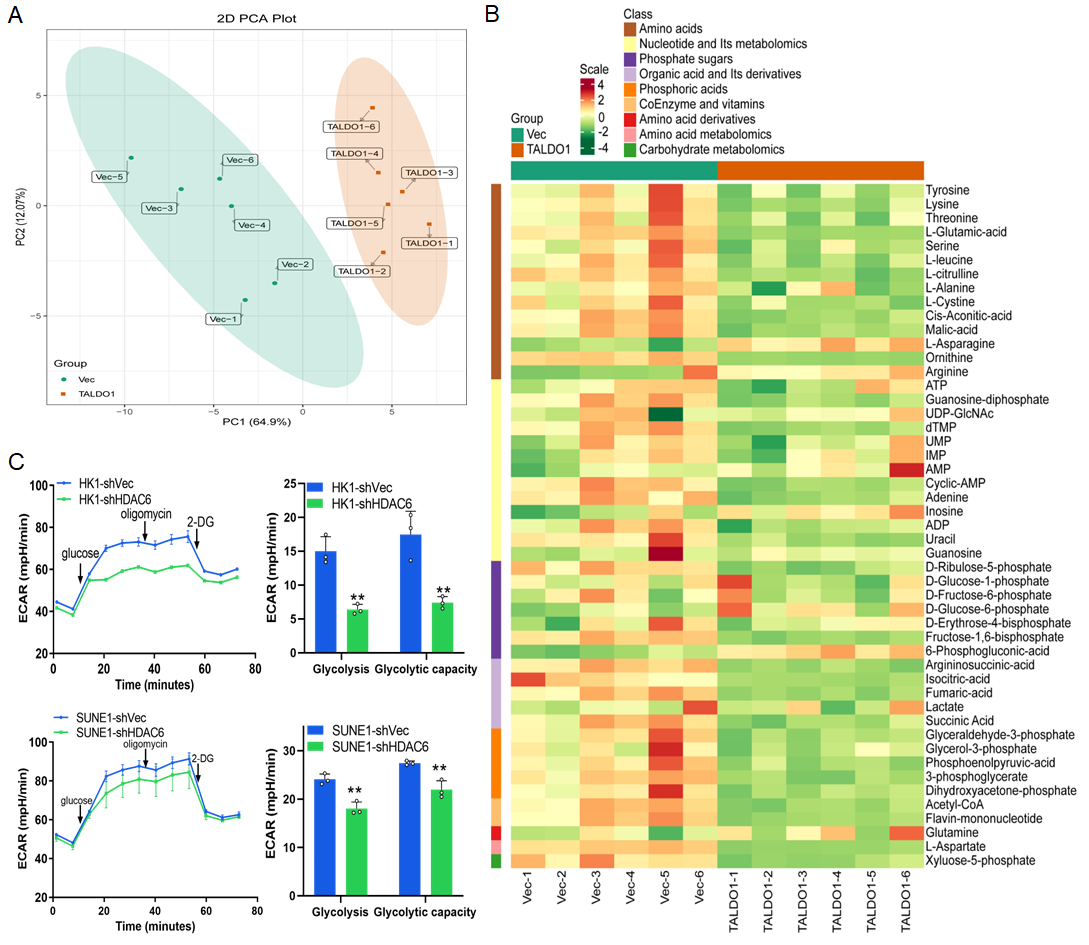
**

**Fig. S10.** **Significant changes in metabolite levels in TALDO1 overexpressing SUNE1 cells. A** Principal Component Analysis (PCA) of targeted metabolites measured in the TALDO1 overexpression group and the control group (n=6). **B** Heatmap of targeted HPLC-MS metabolomics data of TALDO1 overexpression with control group. **C** The extracellular acidification rate (ECAR) of SUNE1-shVec and SUNE1-shHDAC6 stable cells was measured using Seahorse XF assay Kit. Data were shown as the mean ± SD of at least three independent experiments. **P<0.01.


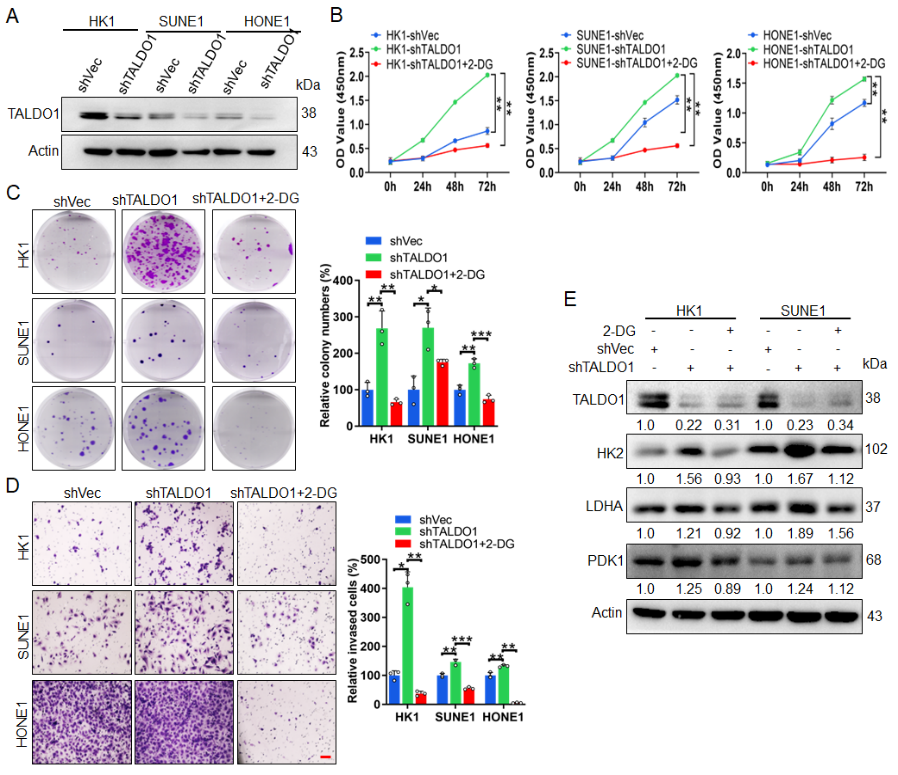


**Fig. S11. TALDO1 inhibited the proliferation and invasion of NPC cells by weakening glycolysis. A** Western blot analysis of TALDO1 expression after stably transfected shTALDO1 or shVec in HK1, SUNE1 and HONE1 cells. **B-E** After treated with glycolysis inhibitor 2-DG (2.5mM, 24h) in TALDO1 stable knockdown NPC cells, CCK-8 assay (**B**) and colony forming assay (**C**) were performed to examine the cell proliferation, Transwell assays was carried out to detect cell invasion (**D**). Scale bar, 50μm. **E** Western blot assay was performed to measure the levels of glycolysis-related metabolic enzyme levels. Data were shown as the mean ± SD of at least three independent experiments. *P<0.05 and **P<0.01 and ***P<0.001.

**Supplementary Tables**

**Table S1.** **Clinical characteristics of NPC patients (tissue microarrays)**

| **Characteristics** | **Number of patients (%)** |
| --- | --- |
| **Gender** |  |
| Male | 81（64.29%） |
| Female | 45（35.71%） |
| **Age** |  |
| ≥45 | 96（76.19%） |
| ＜45 | 30（23.81%） |
| **Tumor size (cm)** |  |
| ＜2 | 107（84.92%） |
| ≥2 | 19（15.08%） |
| **T stage** |  |
| T1 | 45（35.71%） |
| T2 | 48（38.10%） |
| T3 | 30（23.81%） |
| T4 | 3（2.38%） |
| **N stage** |  |
| N0 | 87（69.05%） |
| N1 | 36（28.57%） |
| N2 | 3（2.38 %） |
| N3 | 0（0%） |
| **M stage** |  |
| M0 | 102（80.95%） |
| M1 | 24（19.05%） |
| **Clinical Stage** |  |
| I | 35（27.78%） |
| II | 37（29.37%） |
| III | 25（19.84%） |
| Ⅳ | 29（23.02%） |
| **Metastasis** | 76（60.32%） |
| **Non-Metastasis** | 50（39.68%） |

**Table S2. Clinical data of NPC patients (serum samples)**

| **Characteristics** | **Non-metastatic (%)** | **Metastatic (%)** |
| --- | --- | --- |
| **Gender** |  |  |
| Male | 27（65.85%） | 30（73.17%） |
| Female | 14（34.15%） | 11（26.83%） |
| **Age** |  |  |
| ≥45 | 26（63.41%） | 33（80.49%） |
| ＜45 | 15（36.59%） | 8（19.51%） |
| **T stage** |  |  |
| T1 | 2（4.88%） | 1（2.44%） |
| T2 | 7（17.07%） | 6（14.63%） |
| T3 | 18（43.90%） | 14（34.15%） |
| T4 | 14（34.15%） | 20（48.78%） |
| **N stage** |  |  |
| N0 | 3（7.32%） | 1（2.44%） |
| N1 | 16（39.02%） | 8（19.51%） |
| N2 | 16（39.02%） | 13（31.71%） |
| N3 | 6（14.63%） | 20（48.78%） |
| **M stage** |  |  |
| M0 | 41（100%） | 0（0%） |
| M1 | 0（0%） | 41（100%） |
| **Clinical Stage** |  |  |
| I | 1（2.44%） | 0（0%） |
| II | 3（7.32%） | 0（0%） |
| III | 19（46.34%） | 2（4.88%） |
| Ⅳ | 18（43.90%） | 39（95.12%） |
| **Pathological type** |  |  |
| Undifferentiated non-keratinizing carcinoma | 29（70.73%） | 18（43.90%） |
| Differentiated non-keratinizing carcinoma | 12（29.27%） | 23（56.10%） |

**Table S3** **Oligonucleotide sequences**

**S3-1 Primers for** **construction of recombinant expression plasmids**

| ID | Sequence (5’-3’) |
| --- | --- |
| pcDNA3.1-TALDO1-F | tagtccagtgtggtggaattcATGTCGAGCTCACCCGTGAA |
| pcDNA3.1-TALDO1-R | gccctctagactcgagcggccgcCTACTTTCCATTCTCTGCATTGAACA |
| pcDNA3.1-TALDO1-Flag-F | tagtccagtgtggtggaattcATGTCGAGCTCACCCGTGAA |
| pcDNA3.1-TALDO1-Flag-R | gccctctagactcgagcggccgcCTACTTGTCATCGTCATCCTTGTAGTC |
| pCDH-CMV-TALDO1-F | attcgaatttaaatcggatccATGTCGAGCTCACCCGTGAA |
| pCDH-CMV-TALDO1-R | atggtctttgtagtcggatccCTTTCCATTCTCTGCATTGAACAT |
| pcDNA3.1-HDAC4-His-F | ccgctcgagtctagagggcccATGAGCTCCCAAAGCCATCC |
| pcDNA3.1-HDAC4-His-R | atggtgatgatggaagggcccCAGGGGCGGCTCCTCTTC |
| pcDNA3.1-HDAC5-His-F | ccgctcgagtctagagggcccATGAACTCTCCCAACGAGTCGG |
| pcDNA3.1-HDAC5-His-R | atggtgatgatggaagggcccCAGGGCAGGCTCCTGCTC |
| pcDNA3.1-HDAC6-His-F | ccgctcgagtctagagggcccATGACCTCAACCGGCCAGG |
| pcDNA3.1-HDAC6-His-R | atggtgatgatggaagggcccGTGTGGGTGGGGCATATCC |
| pcDNA3.1-HDAC6-DN-His-F | ccgctcgagtctagagggcccATGACCTCAACCGGCCAGG |
| pcDNA3.1-HDAC6-DN-His-R | atggtgatgatggaagggcccGTGTGGGTGGGGCATATCC |
| pcDNA3.1-SMURF1-His-F | cttggtaccgagctcggatccATGTCGAACCCCGGGACA |
| pcDNA3.1-SMURF1-His-R | atggtgatgatggaagggcccCTCCACAGCAAACCCGCA |
| pcDNA3.1-SYVN1-His-F | ccgctcgagtctagagggcccATGTTCCGCACGGCAGTG |
| pcDNA3.1-SYVN1-His-R | atggtgatgatggaagggcccGTGGGCAACAGGAGACTCCA |
| pGL3-HK2-F | atctgcgatctaagtaagcttGTTAGCTAGGATGGTCTCGATCTCC |
| pGL3-HK2-R | cagtaccggaatgccaagcttATTTCAAGAGACATGACATTAGCACTG |
| pGL3-LDHA-F | atctgcgatctaagtaagcttCGGGCTAGGCAATGGAATC |
| pGL3-LDHA-R | cagtaccggaatgccaagcttTGCTTCTGGAAAGCGGCTC |
| pGL3-PDK1-F | atctgcgatctaagtaagcttTGCCCCTCGGGCACTCAG |
| pGL3-PDK1-R | cagtaccggaatgccaagcttAGCCAGTACGCCAGGTTTCC |
| pGEX-4T-1-TALDO1-F | ccgcgtggatccccggaattcATGTCGAGCTCACCCGTGAA |
| pGEX-4T-1-TALDO1-R | ctcgagtcgacccgggaattcCTTTCCATTCTCTGCATTGAACAT |

**S3-2 Primers for construction of mutated plasmids**

| ID | Sequence (5’-3’) |
| --- | --- |
| TALDO1(K7R)-F | TCACCCGTGAGGCGTCAGAGGATGGAGTCCGCGCTGGACCAG |
| TALDO1(K7R)-R | CCTCTGACGCCTCACGGGTGAGCTCGACATgAATTCCACCAC |
| TALDO1(K7Q)-F | CTCACCCGTGCAGCGTCAGAGGATGGAGTCCGCGCTGGACCA |
| TALDO1(K7Q)-R | CTCTGACGCTGCACGGGTGAGCTCGACATgAATTCCACCACA |
| p300-DY(D1399Y)-F | ATCTTACCTCTATAGTGTTCATTTCTTCCGTCCTAAATGCTT |
| p300-DY(D1399Y)-R | TGAACACTATAGAGGTAAGATATGTATACTCTCCTCTGGTTG |
| Ub-K48R-F | TTTGCTGGGAGGCAGCTGGAAGATGGACGCACCCTGTCTGAC |
| Ub-K48R-R | TTCCAGCTGCCTCCCAGCAAAGATCAACCTCTGCTGGTCAGG |
| Ub-K63R-F | AACATCCAGAGGGAGTCCACCCTGCACCTGGTCCTCCGTCTC |
| Ub-K63R-R | GGTGGACTCCCTCTGGATGTTGTAGTCAGACAGGGTGCGTCC |

**S3-3 shRNA sequences**

| ID | Sequence (5’-3’) |
| --- | --- |
| shTALDO1#1-F | GATCC CTACAACTACTACAAGAAGTTTTCAAGAGAAACTTCTTGTAGTAGTTGTAGTTTTTTG |
| shTALDO1#1-R | AATTCAAAAAACTACAACTACTACAAGAAGTTTCTCTTGAAAACTTCTTGTAGTAGTTGTAGG |
| shTALDO1#2-F | GATCCCTGCAACATGACGTTACTCTTTTCAAGAGAAAGAGTAACGTCATGTTGCAGTTTTTTG |
| shTALDO1#2-R | AATTCAAAAAACTGCAACATGACGTTACTCTTTCTCTTGAAAAGAGTAACGTCATGTTGCAGG |
| shHDAC6#1-F | GATCCCTGCAAGGGATGGATCTGAACTTCAAGAGAGTTCAGATCCATCCCTTGCAGTTTTTTG |
| shHDAC6#1-R | AATTCAAAAAACTGCAAGGGATGGATCTGAACTCTCTTGAAGTTCAGATCCATCCCTTGCAGG |
| shHDAC6#2-F | GATCCCGGTAATGGAACTCAGCACATTTCAAGAGAATGTGCTGAGTTCCATTACCGTTTTTTG |
| shHDAC6#2-R | AATTCAAAAAACGGTAATGGAACTCAGCACATTCTCTTGAAATGTGCTGAGTTCCATTACCGG |
| shMyc-F | CCGGCCTGAGACAGATCAGCAACAACTCGAGTTGTTGCTGATCTGTCTCAGGTTTTTG |
| shMyc-R | AATTCAAAAACCTGAGACAGATCAGCAACAACTCGAGTTGTTGCTGATCTGTCTCAGG |
| shBRCA1-F | CCGGGAGTATGCAAACAGCTATAATCTCGAGATTATAGCTGTTTGCATACTCTTTTTG |
| shBRCA1-R | AATTCAAAAAGAGTATGCAAACAGCTATAATCTCGAGATTATAGCTGTTTGCATACTC |

**S3-4 siRNA sequences**

| ID | Sequence (5’-3’) |
| --- | --- |
| siHDAC4 | GGGAATGTACGACGCCAAA |
| siHDAC5 | CAACGGGAACTTCTTTCCA |

**S3-5 Primers for qPCR**

| ID | Sequence (5’-3’) |
| --- | --- |
| TALDO1-F | CTCACCCGTGAAGCGTCAG |
| TALDO1-R | GTTGGTGGTAGCATCCTGGG |
| HDAC6-F | AAGAAGACCTAATCGTGGGACT |
| HDAC6-R | GCTGTGAACCAACATCAGCTC |
| HK2-F | GAGCCACCACTCACCCTACT |
| HK2-R | CCAGGCATTCGGCAATGTG |
| LDHA-F | ATGGCAACTCTAAAGGATCAGC |
| LDHA-R | CCAACCCCAACAACTGTAATCT |
| PDK1-F | CTGTGATACGGATCAGAAACCG |
| PDK1-R | TCCACCAAACAATAAAGAGTGCT |
| ACTIN-F | CATGTACGTTGCTATCCAGGC |
| ACTIN-R | CTCCTTAATGTCACGCACGAT |

**Table S4** **Acetylated metabolism enzymes in LBH589 treated HK1 cells (LBH/DMSO>1.5, P value<0.05)**

| **Protein accession** | **Gene name** | **DMSO_1** | **DMSO_2** | **DMSO_3** | **LBH_1** | **LBH_2** | **LBH_3** | **LBH/Ctrl Ratio** | **LBH/Ctrl P value** |
| --- | --- | --- | --- | --- | --- | --- | --- | --- | --- |
| P37837 | TALDO1 | 0.271 | 0.25 | 0.299 | 1.571 | 1.572 | 1.552 | 5.726 | 0.000881 |
| P09622 | DLD | 0.569 | 0.519 | 0.599 | 1.345 | 1.354 | 1.332 | 2.389 | 0.002325 |
| P40939 | HADHA | 0.563 | 0.573 | 0.582 | 1.329 | 1.342 | 1.335 | 2.332 | 5.81E-08 |
| O95573 | ACSL3 | 0.692 | 0.668 | 0.572 | 1.836 | 1.079 | 0.907 | 1.978 | 0.043998 |
| P05023 | ATP1A1 | 0.637 | 0.639 | 0.697 | 1.342 | 1.273 | 1.192 | 1.93 | 0.000135 |
| P07954 | FH | 0.698 | 0.743 | 0.679 | 1.182 | 1.278 | 1.237 | 1.744 | 9.56E-05 |
| P16152 | CBR1 | 0.727 | 0.594 | 0.805 | 1.213 | 1.21 | 1.275 | 1.739 | 0.003501 |
| P19367 | HK1 | 0.768 | 0.715 | 0.677 | 1.206 | 1.215 | 1.245 | 1.697 | 0.000145 |
| P40926 | MDH2 | 0.756 | 0.74 | 0.69 | 1.19 | 1.201 | 1.254 | 1.667 | 8.45E-05 |
| Q9P0J1 | PDP1 | 0.724 | 0.795 | 0.737 | 1.169 | 1.3 | 1.123 | 1.592 | 0.000884 |
| P04843 | RPN1 | 0.781 | 0.648 | 0.829 | 1.184 | 1.202 | 1.208 | 1.592 | 0.024162 |
| O75947 | ATP5PD | 0.803 | 0.756 | 0.702 | 1.165 | 1.254 | 1.171 | 1.588 | 0.000523 |
| Q9P2R7 | SUCLA2 | 0.77 | 0.733 | 0.799 | 1.113 | 1.157 | 1.289 | 1.546 | 0.001003 |
| P35914 | HMGCL | 0.755 | 0.784 | 0.785 | 1.121 | 1.22 | 1.201 | 1.524 | 0.000135 |
| P29803 | PDHA2 | 0.718 | 0.817 | 0.796 | 1.189 | 1.162 | 1.183 | 1.516 | 0.000479 |
