# Supplementary material for: Deacetylation of TALDO1 by HDAC6 promotes glycolysis and nasopharyngeal carcinoma progression through a moonlighting function
Source: Cell Death Dis. 2025 Oct 21;16(1):743. doi: 10.1038/s41419-025-08057-2 (PMC12540763; doi:10.1038/s41419-025-08057-2)

**Supplementary Materia**

**Uncropped original western blots**

**Deacetylation of TALDO1 by HDAC6 promotes glycolysis and nasopharyngeal carcinoma progression through a moonlighting function**

Xingzhi Peng, Peijun Zhou, Kun Zhang, Likang Chen, Min Tang, Qin Zhou, Janwi Peng, Lifang Yang


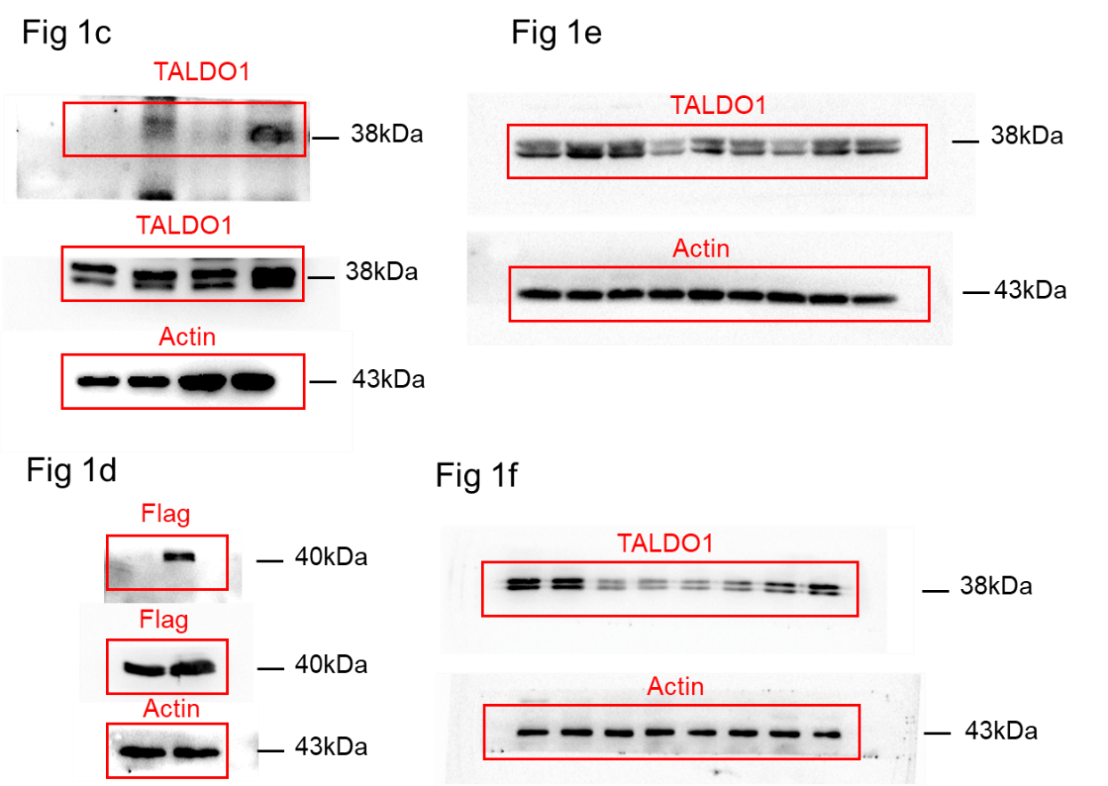


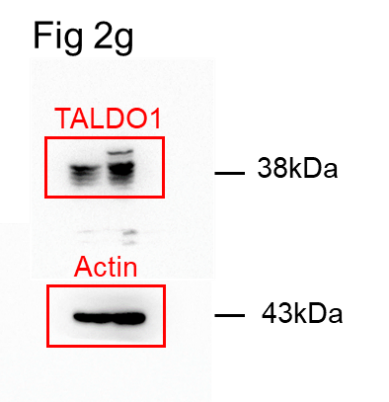


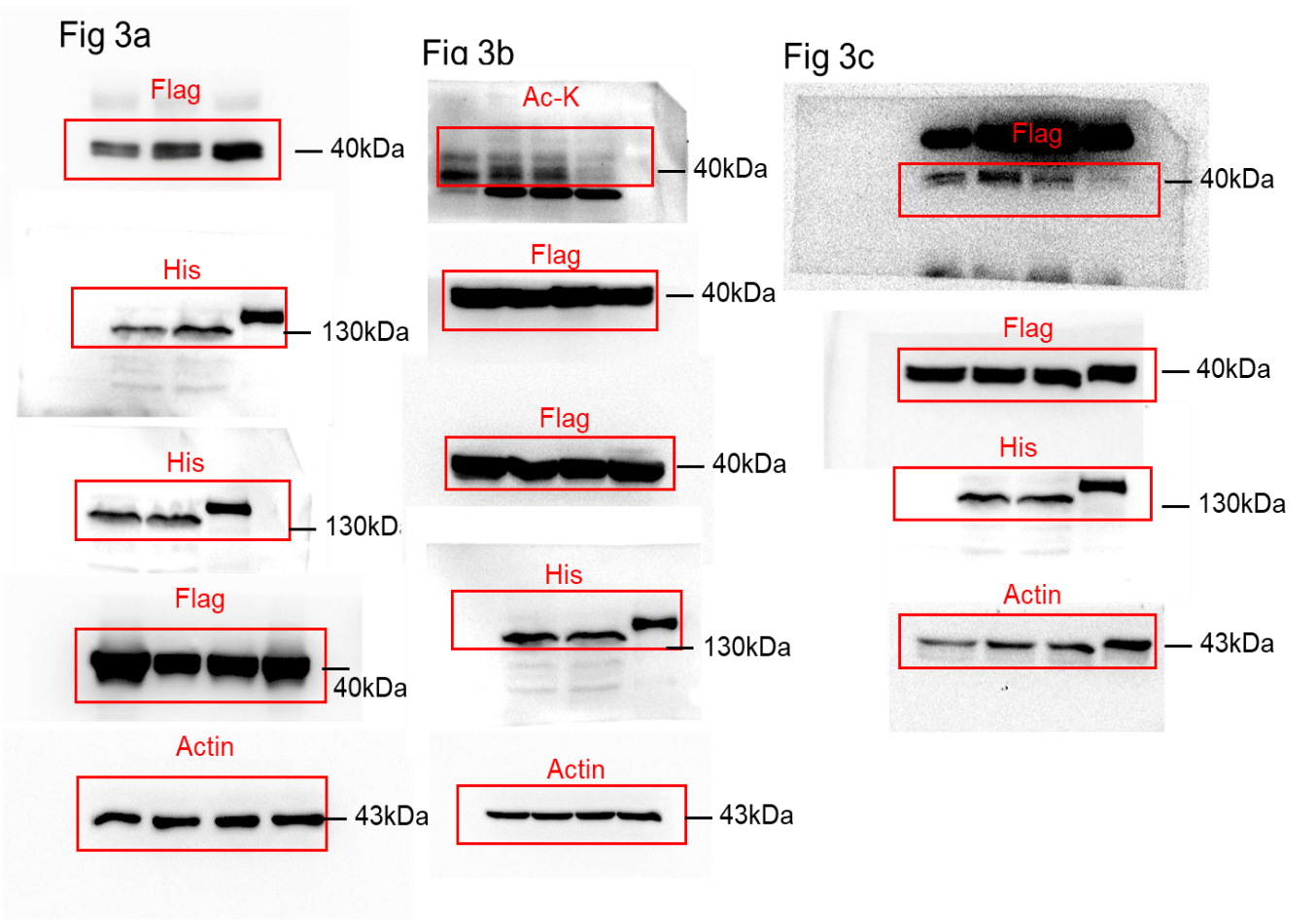


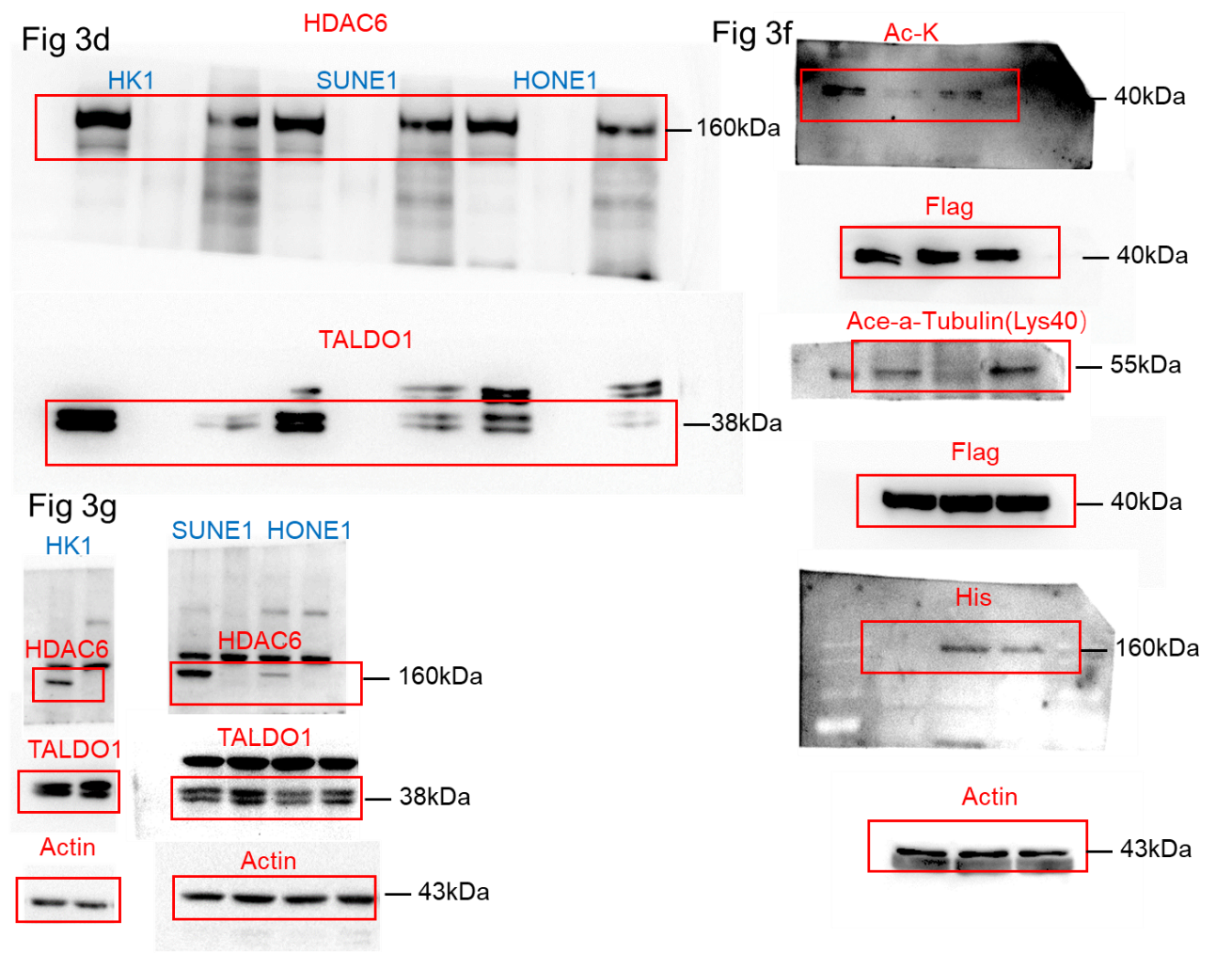


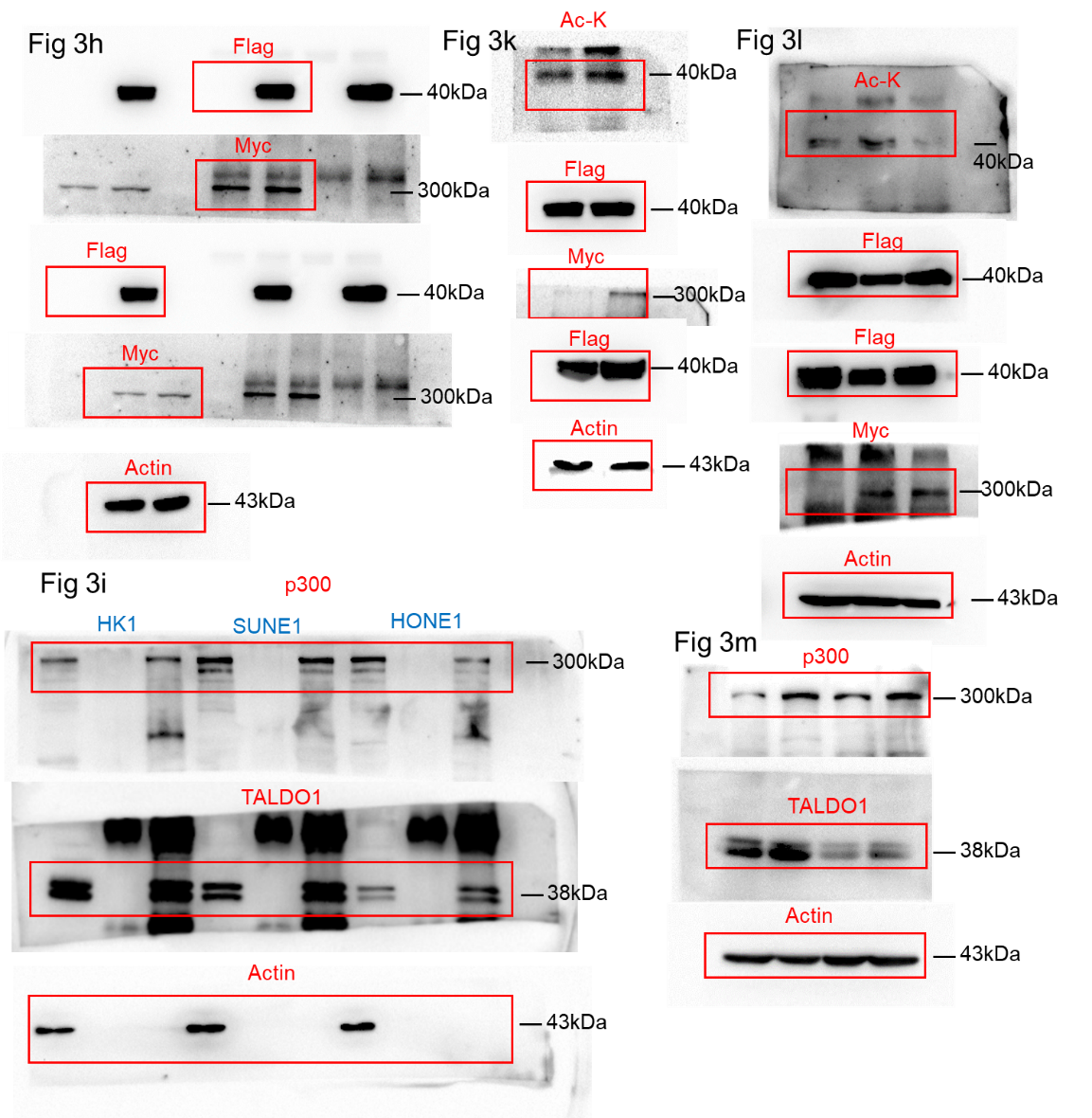


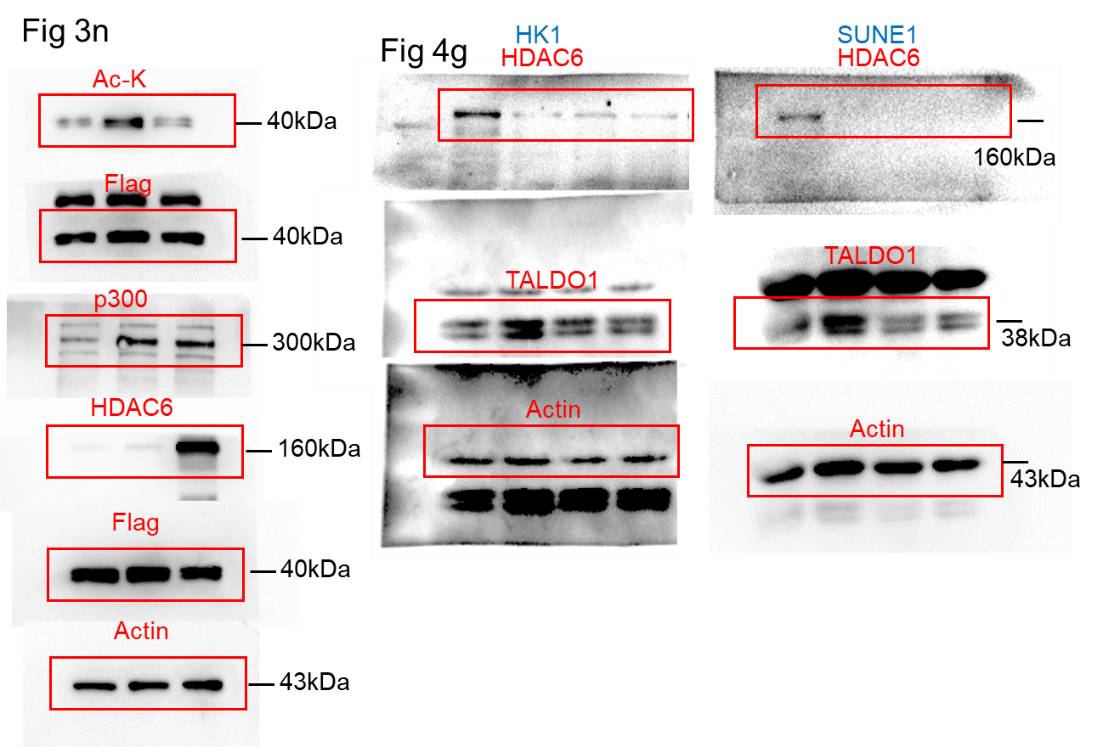


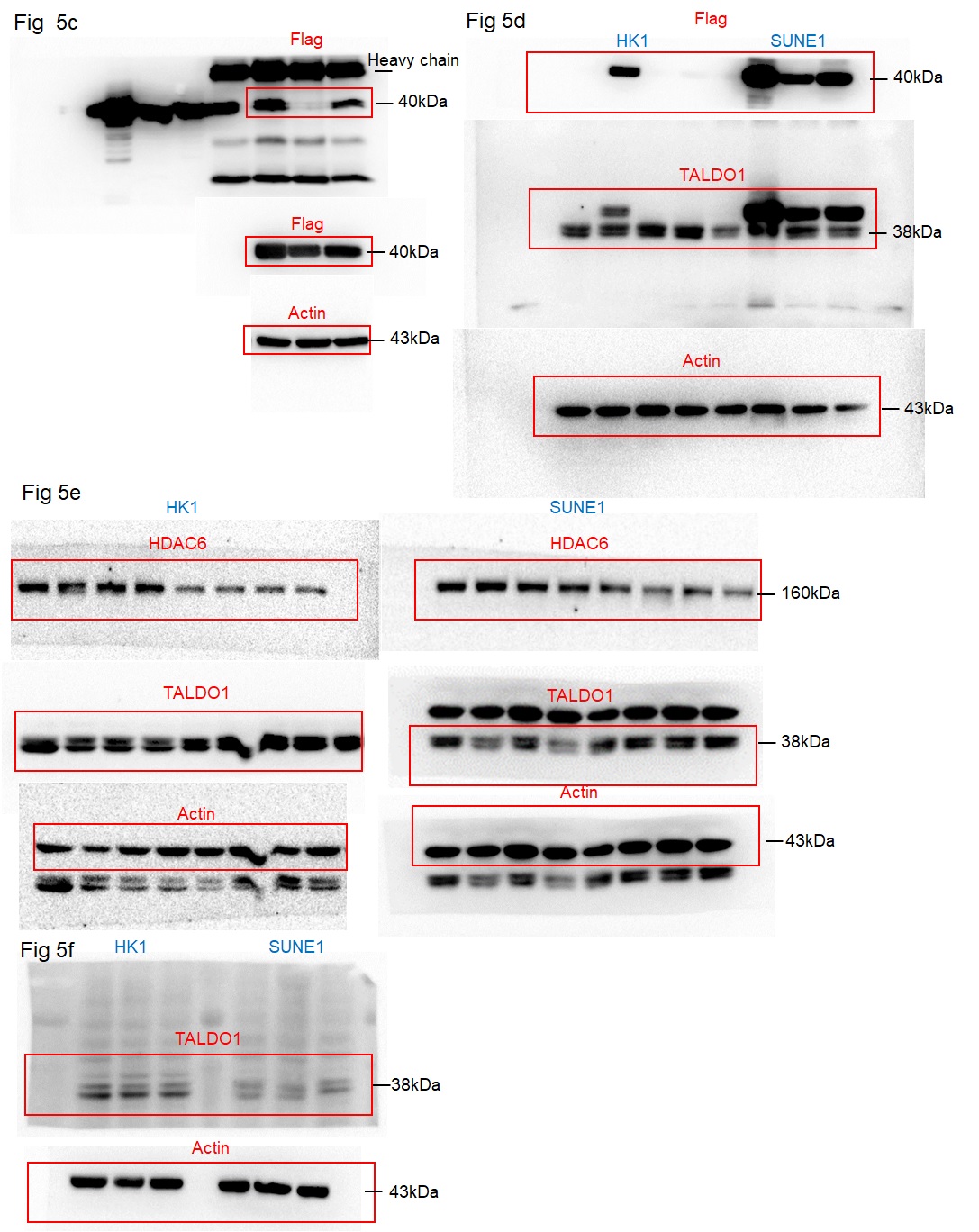


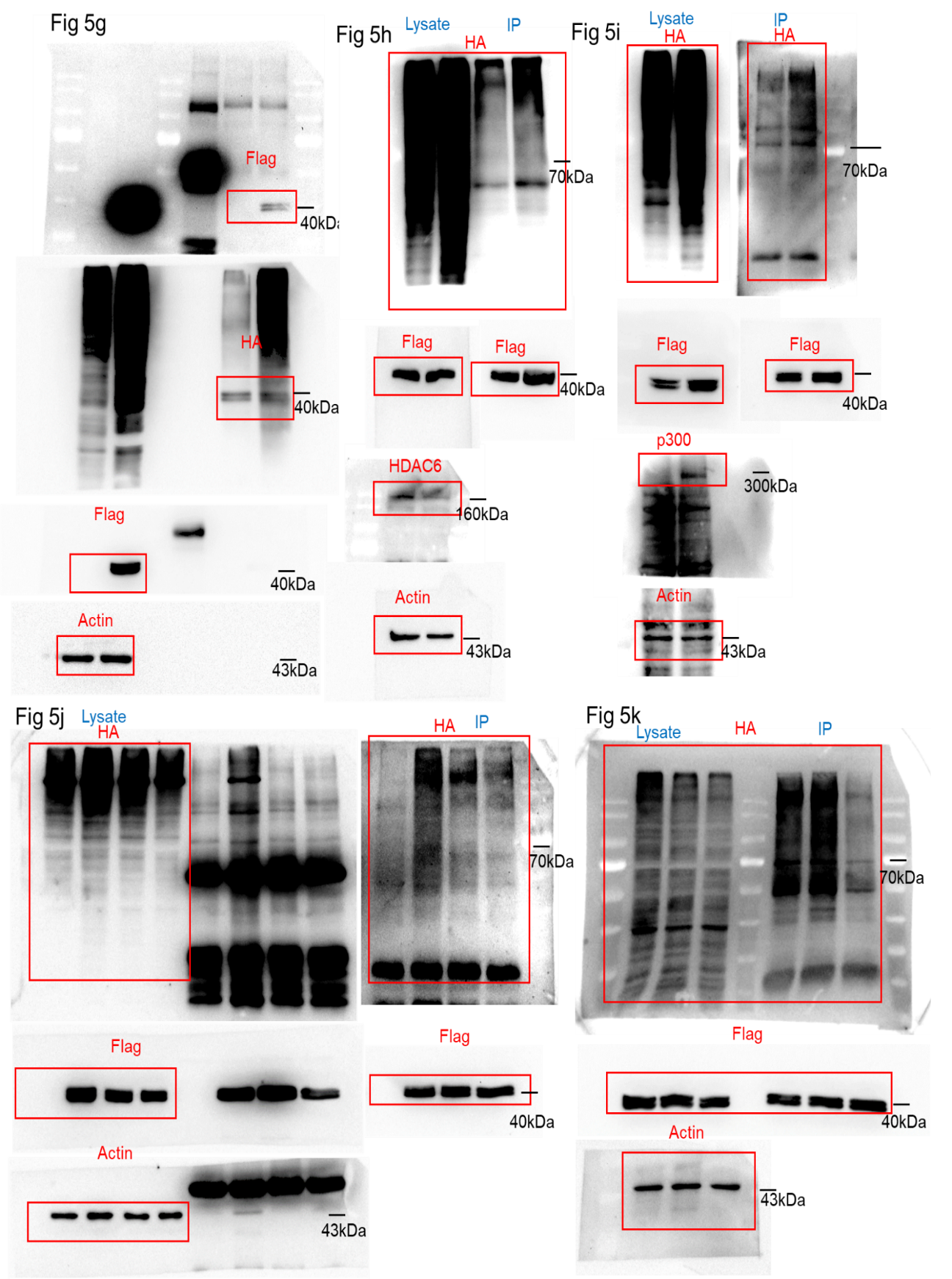


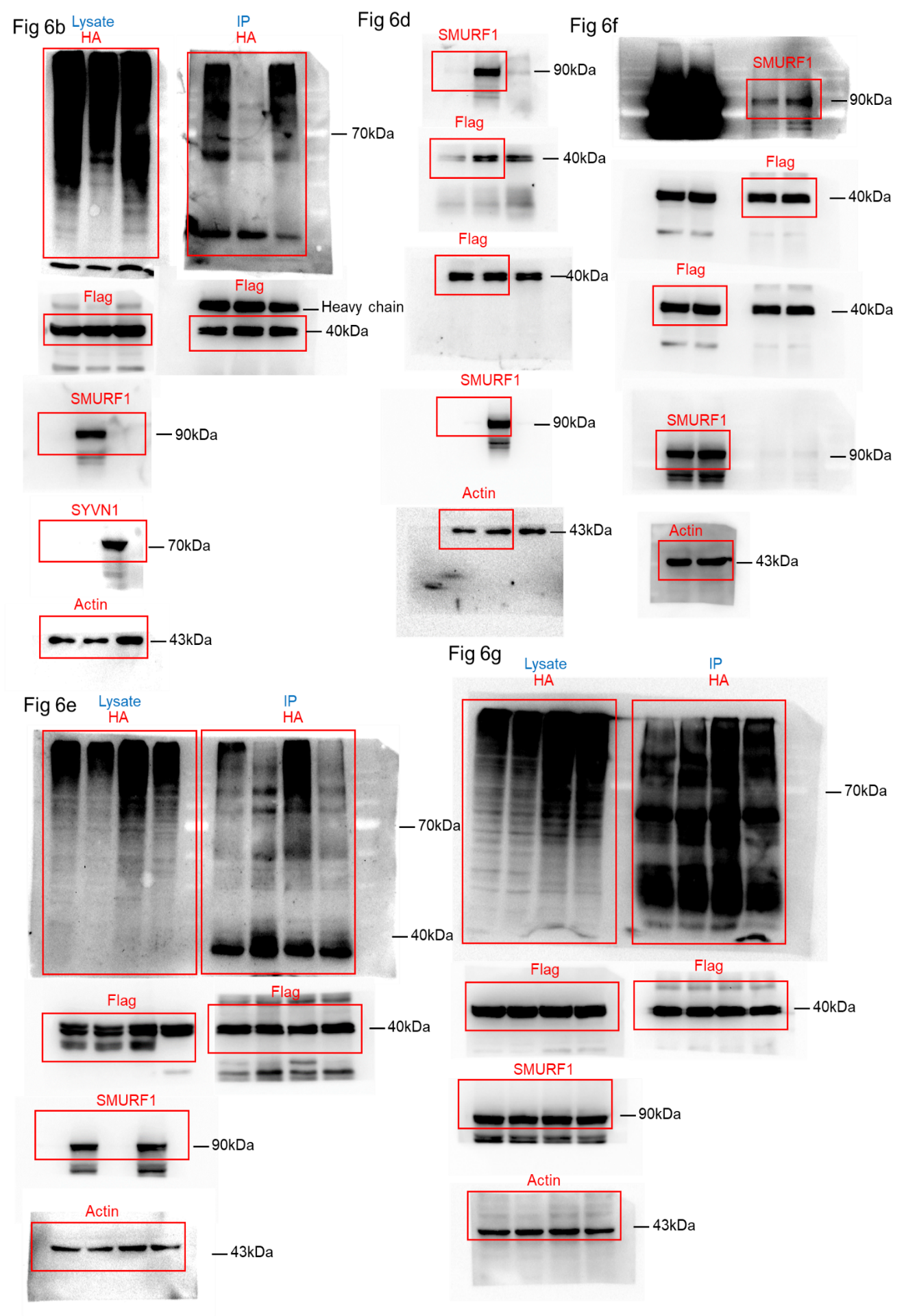


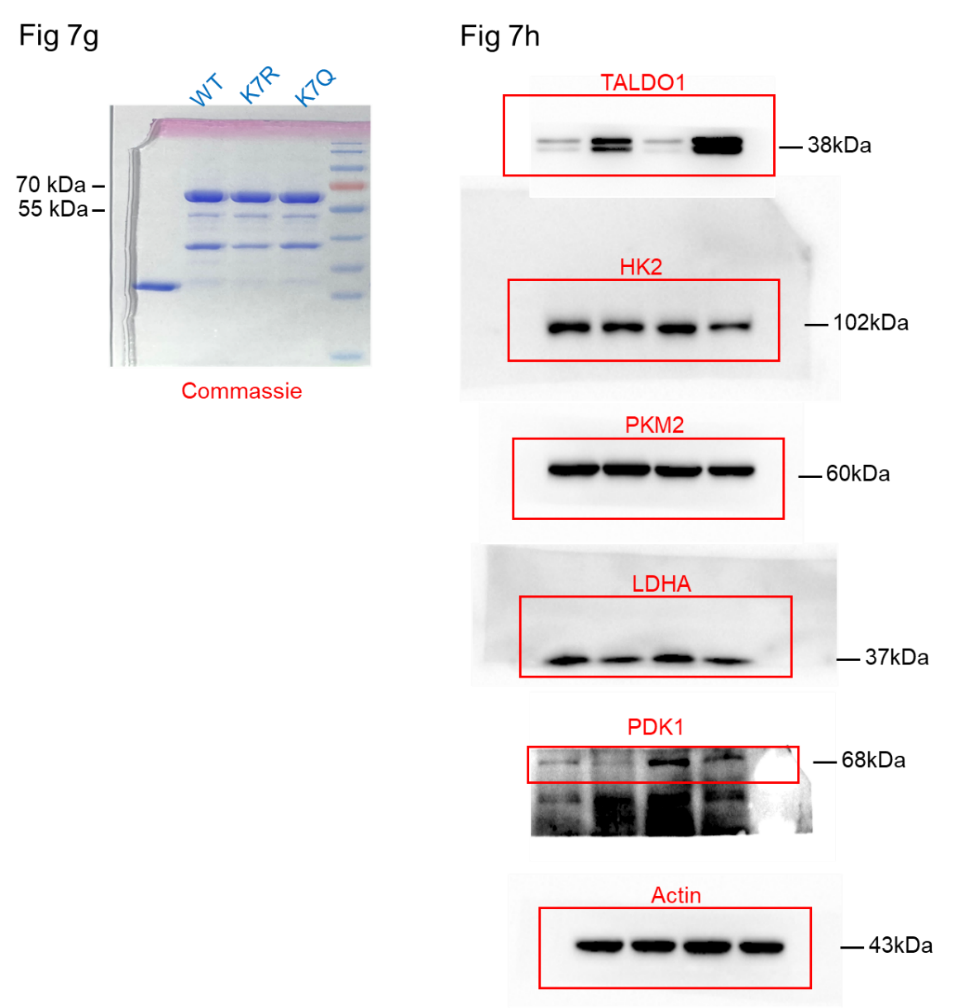


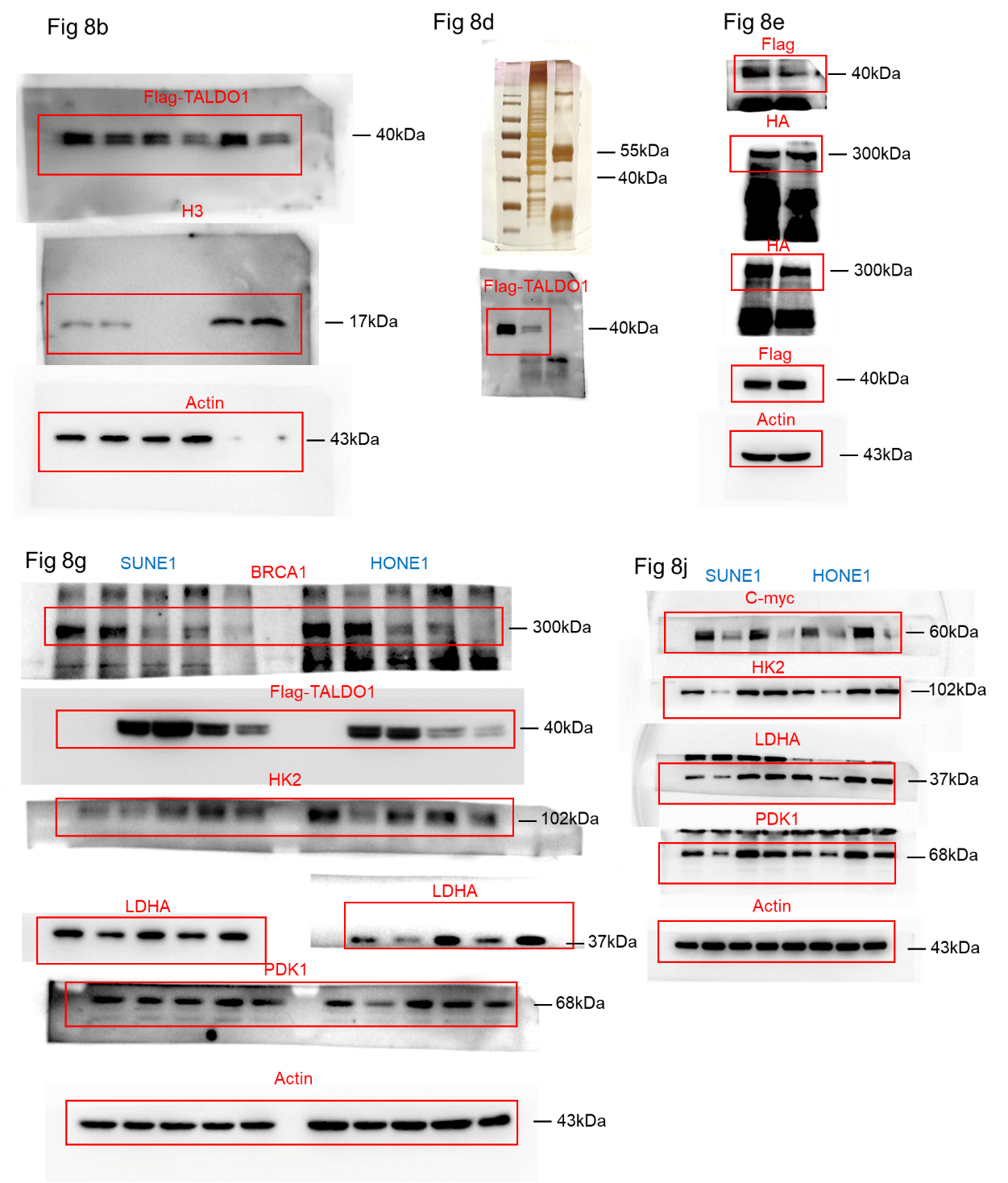


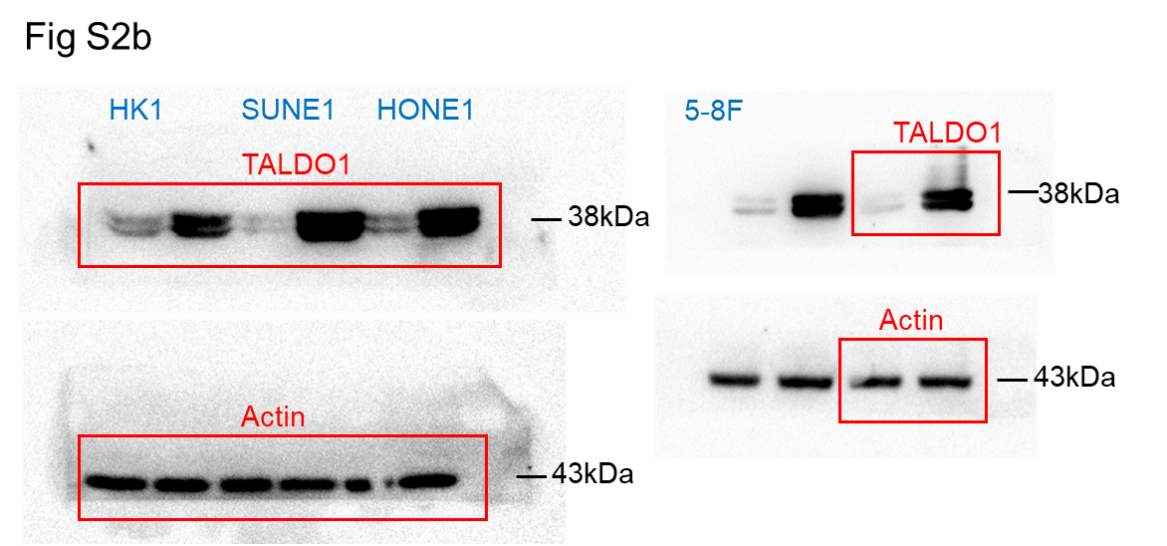


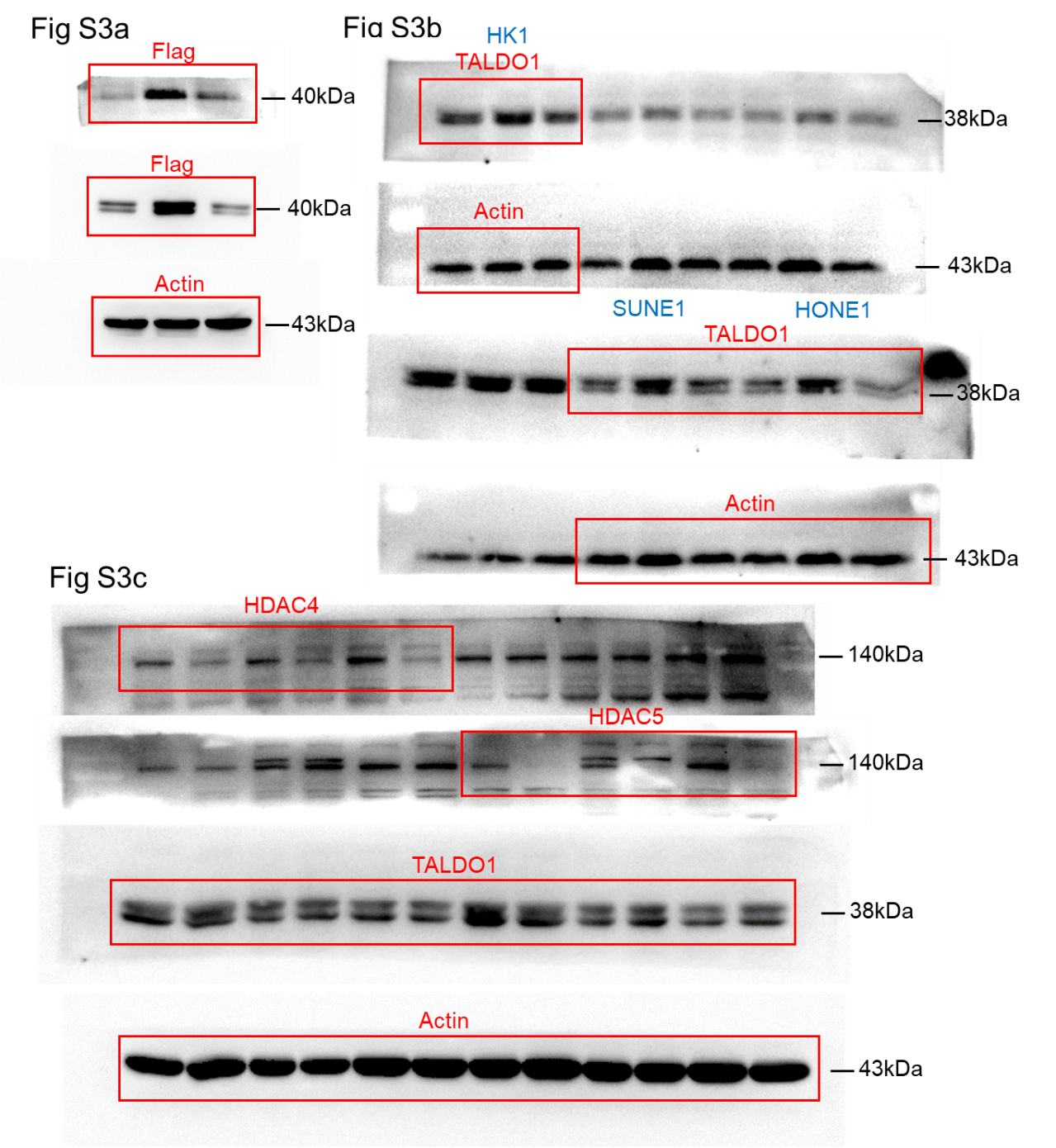


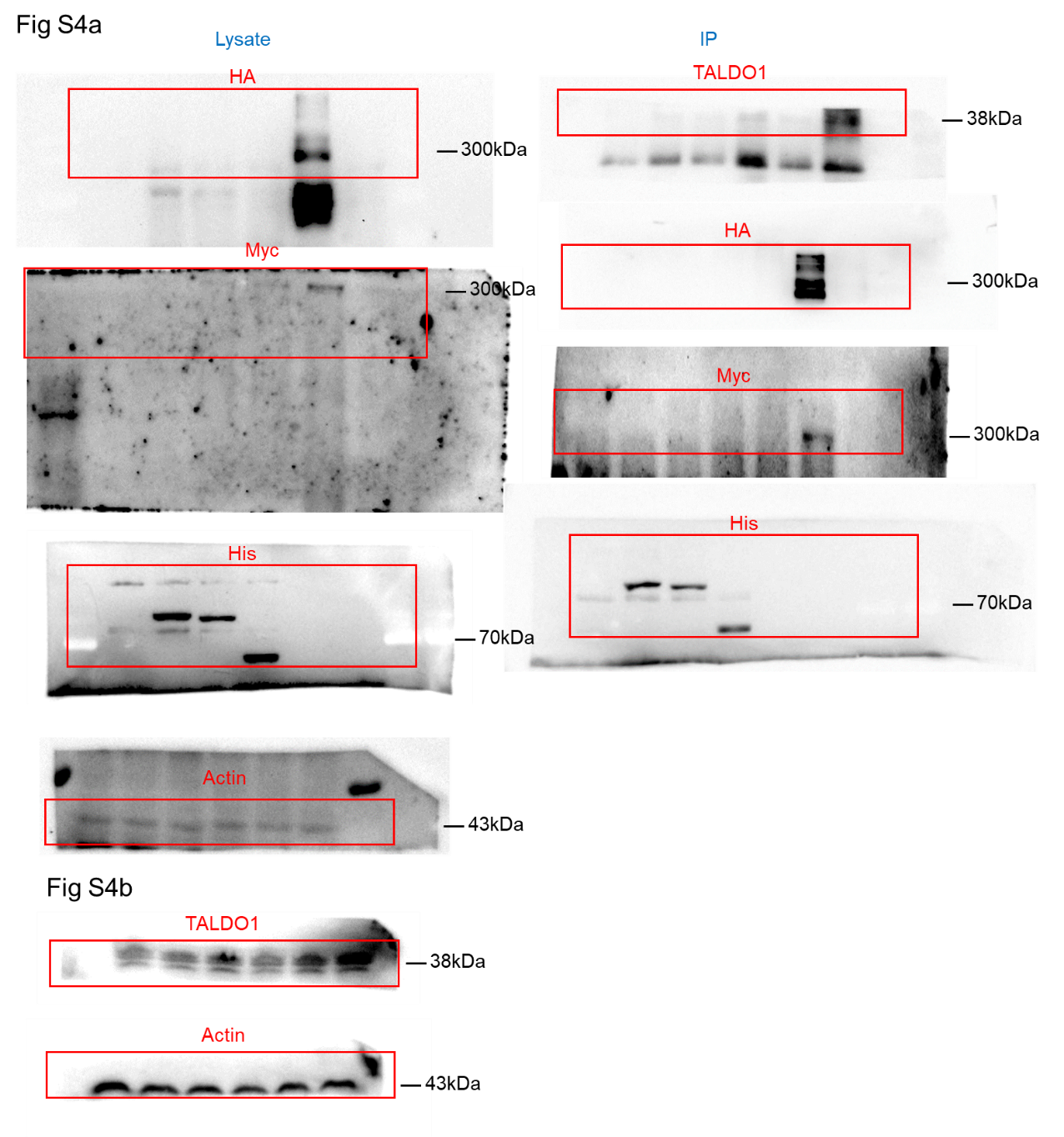


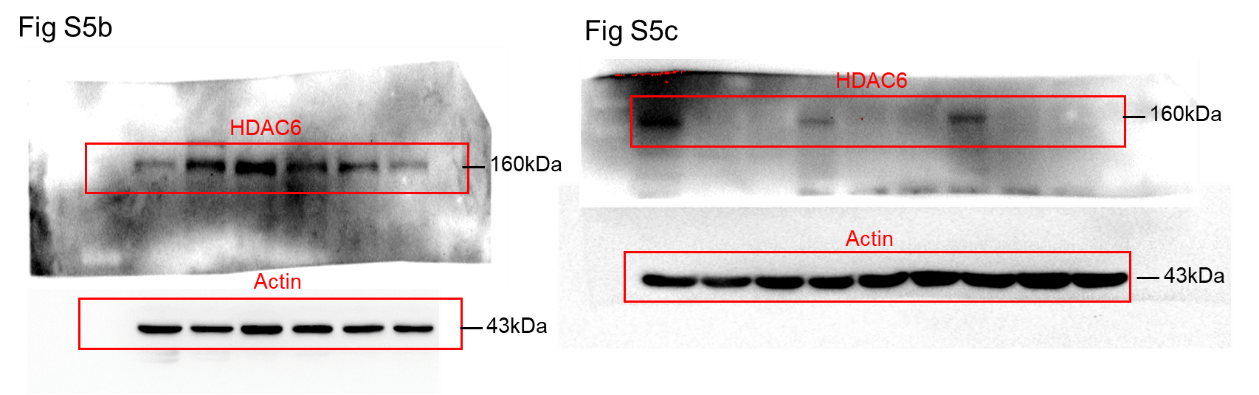


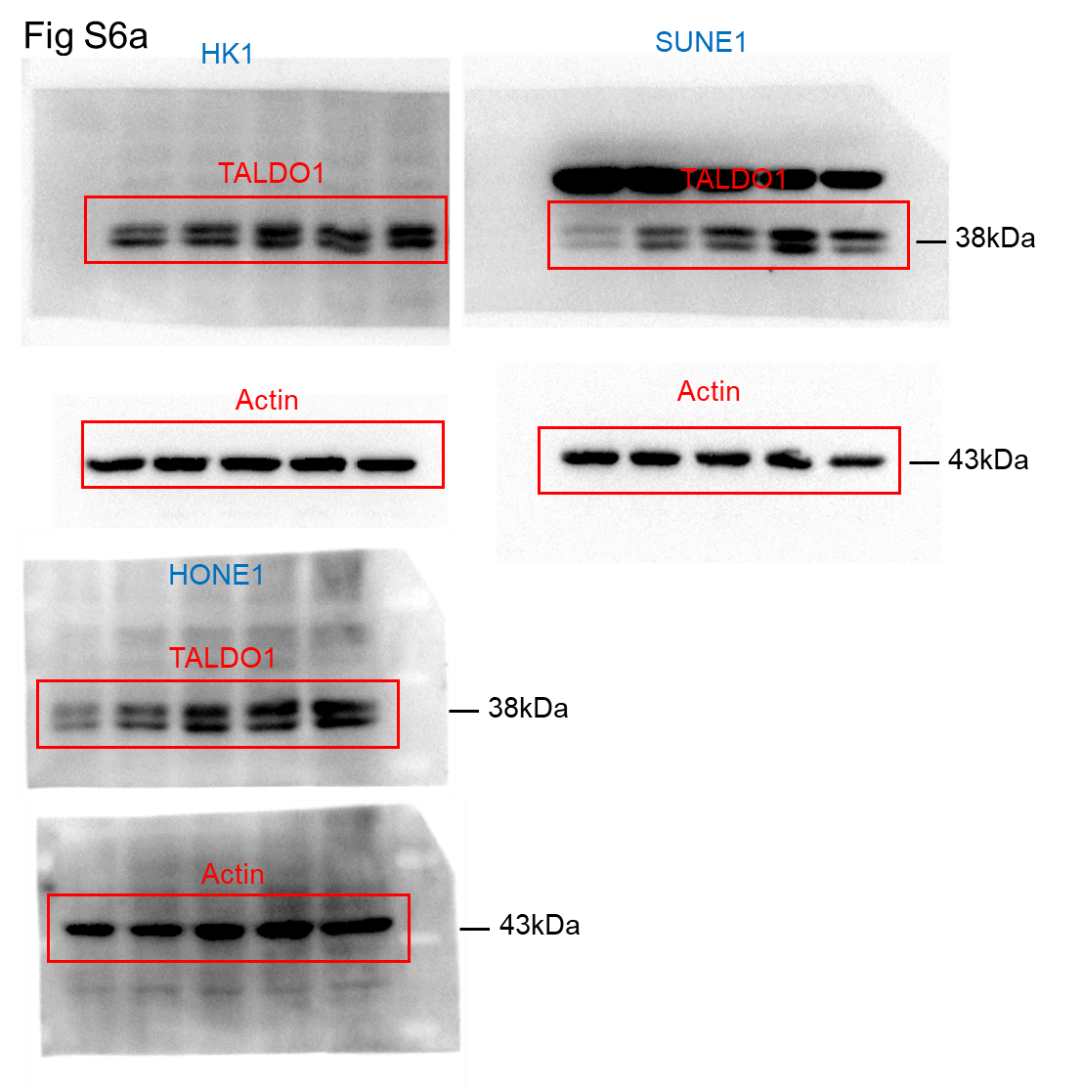


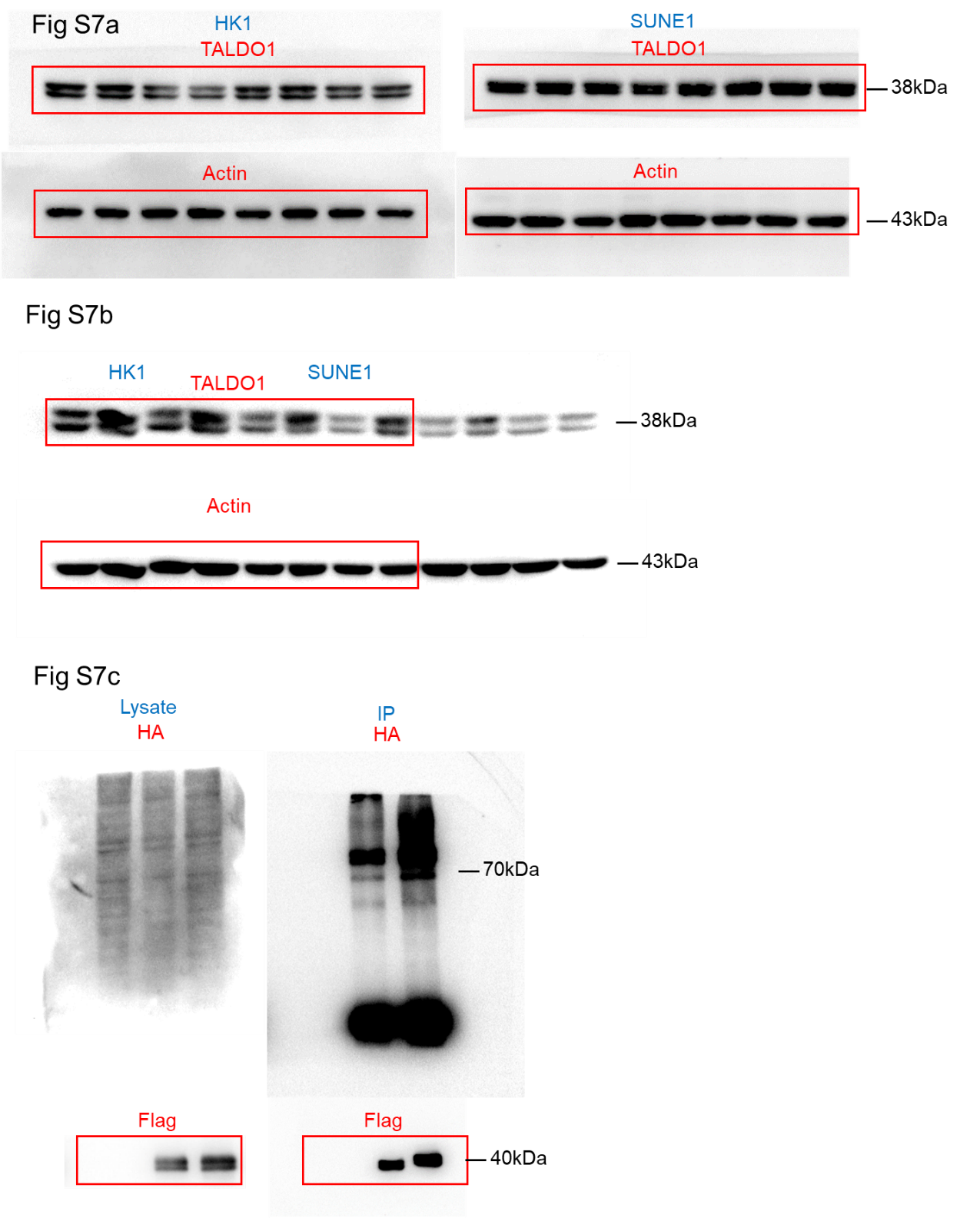


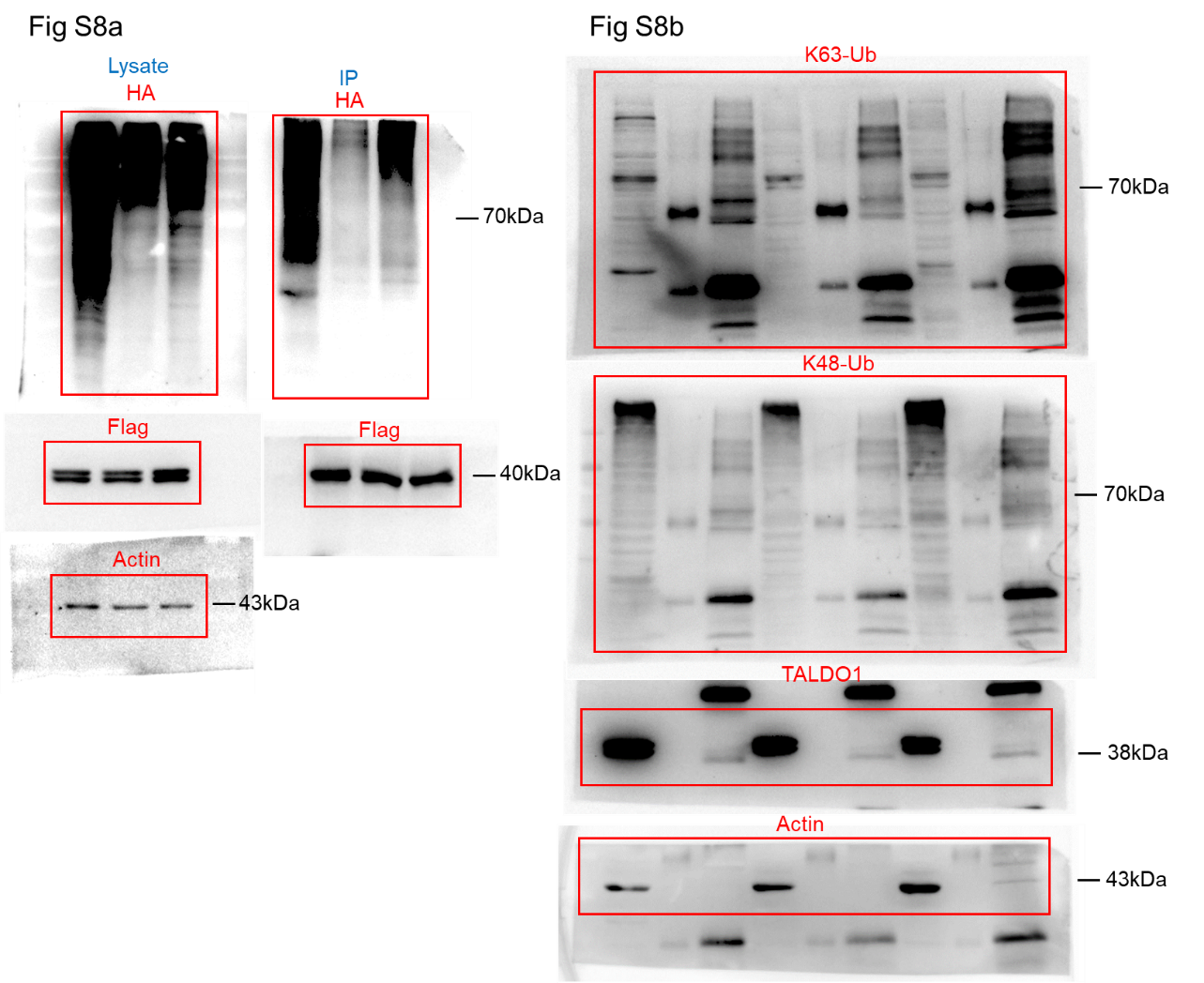


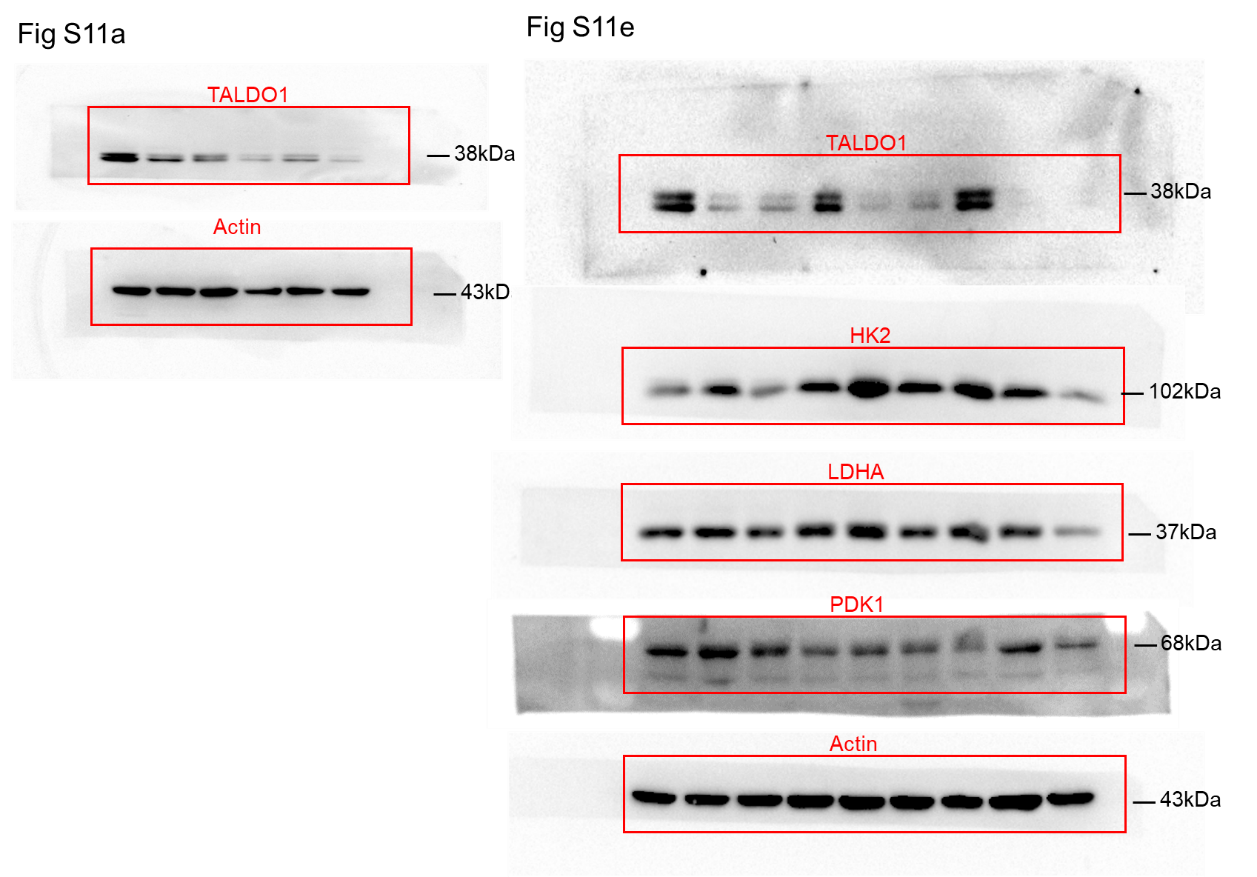

Supplement: Supplementary file 2 — original western-blots [file 41419_2025_8057_MOESM2_ESM.docx]
